# ZNRF1 deficiency disrupts Fas ligand trafficking and immune balance

**DOI:** 10.1038/s41419-026-08566-8

**Published:** 2026-03-28

**Authors:** Ting-Yu Lai, Yung-Chi Chang, You-Sheng Lin, Ching-Yi Tsai, Pu Ou-Yang, Chien-Chia Chen, Meng-Kun Tsai, Li-Chung Hsu, Chih-Yuan Lee

**Affiliations:** 1 Department of Surgery, National Taiwan University Hospital, Taipei, Taiwan, ROC; 2 Institute of Molecular Medicine, College of Medicine, National Taiwan University, Taipei, Taiwan, ROC; 3 Department of Medical Research, National Taiwan University Hospital, Taipei, Taiwan, ROC; 4 Division of General Surgery, Department of Surgery, National Taiwan University Biomedical Park Hospital, National Taiwan University Hospital Hsinchu Branch, Hsinchu, Taiwan, ROC; 5 Graduate Institute of Immunology, College of Medicine, National Taiwan University, Taipei, Taiwan, ROC

**Keywords:** Cell biology, Cell death and immune response

## Abstract

Fas ligand (FasL)-mediated apoptosis constrains immune responses by eliminating activated lymphocytes, yet how FasL is delivered to the plasma membrane in myeloid cells remains unclear. We identify the RING E3 ligase ZNRF1 as a macrophage-intrinsic checkpoint that licenses terminal trafficking and surface exposure of FasL. Myeloid-specific *Znrf1* deletion caused age-dependent spontaneous splenomegaly and, upon allosensitization, elevated CD4^+^/CD8^+^ T-cell ratios, enlarged germinal centers with heightened IL-21/Tfh activity, and augmented alloantibody production. In macrophages, ZNRF1 deficiency disrupted the link between total and surface FasL. Although cellular FasL levels increased upon stimulation, there was no corresponding elevation in surface FasL, indicating a defect in terminal trafficking or docking. Confocal imaging showed preserved peripheral polarization of LAMP1⁺ lysosome-related organelles while FasL cargo did not co-accumulate at the cortex, indicating a late docking/fusion defect. Biochemically, ZNRF1 deficiency weakened the Munc18-2 (*Stxbp2*)–Syntaxin-3 (S*tx3*) interaction; *Stxbp2* knockdown reduced surface FasL, and reconstitution with wild-type ZNRF1—but not the catalytically inactive C184A mutant—restored surface FasL despite similar complex assembly, establishing a requirement for ZNRF1 E3 activity. Functionally, *Znrf1*-deficient macrophages displayed impaired FasL-dependent killing of activated CD4⁺ T cells and Fas-sensitive targets, not rescued by stronger LPS priming. These findings define a ZNRF1–Munc18-2–Stx3 axis that couples lysosome-related organelle polarization to fusion, ensuring timely FasL availability at the macrophage surface and suggesting a tractable node to modulate immune hyperactivation.

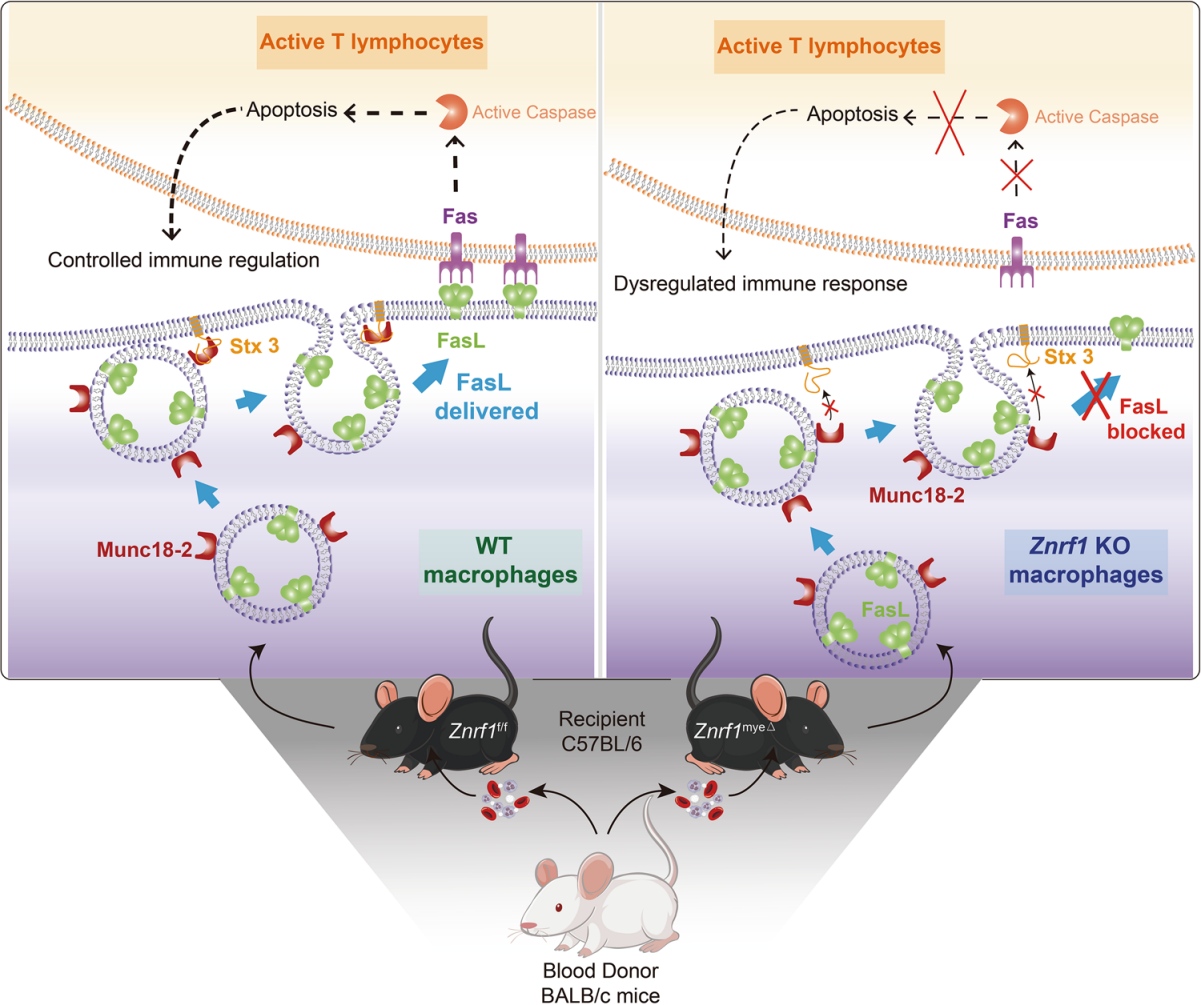

## Introduction

Specialized secretory pathways are a defining feature of immune cells. Many leukocytes—including macrophages, mast cells, lymphocytes, and natural killer (NK) cells—repurpose lysosome-related organelles (LROs) as regulated secretory compartments. These secretory lysosomes contain both lysosomal hydrolases and inducible effector cargos and undergo stimulus-dependent fusion with the plasma membrane to release contents at defined sites [1]. Antigen-presenting cells (APCs) such as macrophages, dendritic cells, and B cells display antigenic peptides on MHC class II and share with secretory lysosomes key features of cargo storage and regulated exocytosis [2]. In parallel, Fc-receptor ligation or chemotactic cues trigger mast cells and basophils to discharge histamine and serotonin from secretory granules, a process mechanistically linked to secretory lysosomes [3]. Likewise, cytotoxic T cells and NK cells kill targets by releasing granules enriched for perforin and granzymes—another LRO-dependent pathway [2, 4].

The transmembrane protein Fas ligand (FasL), a member of the tumor necrosis factor superfamily, is stored in secretory lysosomes within cytotoxic T cells and ocular epithelial cells [1, 5]. The FasL-mediated pathway is a crucial regulator of immune cell apoptosis that is essential to immune system balance and cancer progression [6, 7]. Disruptions in the Fas–FasL system can contribute to lymphoproliferative and autoimmune disorders and facilitate immune evasion by cancer cells [8]. Although FasL is widely capable of inducing apoptosis in Fas-expressing cells, the cellular mechanisms that control FasL availability at the cell surface—particularly its trafficking from intracellular stores—remain incompletely defined.

Macrophages are multifunctional effectors that initiate inflammation, present antigen, clear apoptotic cells, and can directly delete responder lymphocytes by expressing surface FasL. FasL–Fas engagement initiates activation-induced cell death (AICD) in T cells, a dominant homeostatic mechanism that restrains excessive clonal expansion [9, 10]. Despite the importance of macrophages at sites of inflammation, how FasL expression is post-translationally regulated in macrophages is not well understood.

In another study, we demonstrated that zinc and ring finger 1 (ZNRF1)—a ubiquitin E3 ligase—regulates immune responses through Toll-like receptor (TLR) 4 [11]. ZNRF1 localizes to the endosome or lysosome compartment and interacts with β-tubulin type 2, implicating cytoskeletal interfaces in its function [12]. ZNRF1 depletion causes epidermal growth factor receptor accumulation in early endosomes, consistent with a role in endolysosomal sorting [13]. These observations led us to hypothesize that ZNRF1 coordinates late trafficking steps of LRO cargos in macrophages, thereby tuning the availability of key immune effectors at the plasma membrane.

## Materials and methods

### Mice

Conditional *Znrf1*^f/f^ mice on the C57BL/6 background were generated as described [11]. For hematopoietic deletion, *Znrf1*^f/f^ mice were crossed to Mx1-Cre transgenics in which Cre is driven by the interferon-inducible Mx1 promoter [14]. Cre was induced by intraperitoneal poly(I:C) (10 µg/g body weight) administered twice on alternate days at 3–4 weeks of age; mice were analyzed ≥14 days later and are referred to as Mx1-Cre:*Znrf1*^Δ^ (*Znrf1*^Δ^) [14]. For myeloid-specific deletion, *Znrf1*^f/f^ mice were crossed with LysM-Cre transgenics to obtain *Znrf1*^myeΔ^ mice. Unless otherwise indicated, mice were 6–10 weeks old, C57BL/6 background, and age-/sex-matched littermates were used as controls. All animals were maintained under specific-pathogen-free conditions. Procedures followed institutional and national guidelines and were approved by the Institutional Animal Care and Use Committee, College of Medicine, National Taiwan University (IACUC 20201165). Group sizes for animal experiments were selected based on prior experience and published studies while minimizing animal use; the exact n for each experiment is reported in the corresponding figure legends, and n denotes biological replicates (individual mice) unless otherwise stated.

### Cell culture and BMDM preparation

HEK293T, L1210, and RAW264.7 cell lines were obtained from the American Type Culture Collection (ATCC). Cell lines were not recently authenticated (e.g., STR profiling was not performed), but were routinely tested for mycoplasma contamination and found to be negative. HEK293T and L1210 cells were cultured in Dulbecco’s modified Eagle medium (DMEM; Gibco, Thermo Fisher Scientific) supplemented with 10% (v/v) heat-inactivated fetal bovine serum and 100 U/mL penicillin-streptomycin at 37°C in a humidified incubator with 5% CO₂. Murine macrophage-like RAW264.7 cells were maintained in RPMI 1640 solution (Gibco) supplemented with 10% fetal bovine serum. BMDMs were prepared per the procedure described in our earlier study [11]. Femurs and tibias were harvested from mice between 6 and 8 weeks old, and the bone marrow cells were flushed out using a 25-gauge syringe filled with DMEM. The harvested bone marrow cells were cultured in high-glucose DMEM supplemented with 20% L929-cell-conditioned medium for 7 days to induce macrophage differentiation. The differentiated BMDMs were subsequently cultured in DMEM containing 10 ng/mL macrophage colony-stimulating factor.

### Plasmids

The full-length ZNRF1 was amplified using polymerase chain reaction from a mixture of cDNA derived from C57BL/6 mouse livers and cloned onto the pcDNA3 vector (Invitrogen, Carlsbad, CA) containing a C-terminal FLAG epitope tag. A FLAG-tagged ZNRF1(C184A) mutant plasmid was generated using site-directed mutagenesis with a QuickChange II Site-Directed Mutagenesis Kit (Stratagene, La Jolla, CA). Both FLAG-ZNRF1 and FLAG-ZNRF1(C184A) constructs were subsequently cloned onto the pLKO-Tet-On AS2 lentiviral vector (National RNAi Core Facility, Academia Sinica, Taiwan) to produce lentiviruses. FasL cDNA, obtained from Origene (Rockville, MD), was polymerase chain reaction–amplified and cloned into a C-terminally FLAG-tagged pLKO-AS2 lentiviral vector.

### Generation of Znrf1^−/−^ Raw264.7 cells using the CRISPR/Cas9 system

To generate *Znrf1*^−/−^ Raw264.7 cells, HEK293T cells were cotransfected with the lentiviral packaging plasmids pMD.G and pCMVR8.91 and a CRISPR/single guide RNA (sgRNA)/puro expression plasmid encoding an sgRNA sequence targeting mouse ZNRF1. After 48 h, the culture medium containing the lentiviruses was harvested and used to infect Raw264.7 cells for 24 h. The infected cells were subjected to puromycin selection to enrich the transduced cells. The sgRNA target sequences were 5’-GTCCACCTATGCCCACGGCAA-3’ (sgRNA #1) and 5’-GTAGAGACGGGATGCTGTACC-3’ (sgRNA #2).

### Grouping and allogeneic immunization of experimental animals

Male *Znrf1*^f/f^ and *Znrf1*^myeΔ^ mice aged 7–8 weeks were randomly divided into immunized and nonimmunized groups. Mice in the immunized group were injected with 50 µL of whole blood collected from BALB/c mice through the jugular vein. The non-immunized group received an injection of sterile phosphate-buffered saline (PBS). Blood was collected from all mice 2 weeks following immunization, and serum samples were separated and stored at −20 °C.

### Serum anti-BALB/c antibody detection and isotyping by flow cytometry

The presence of anti-BALB/c antibodies in the serum of the nonimmunized and immunized mice was assessed using a flow cytometry assay. In the assay, 2 × 10^5^ BALB/c splenocytes were incubated with 100 µL of serum samples from *Znrf1*^f/f^ or *Znrf1*^myeΔ^ mice, diluted 1:20 in fluorescence-activated cell sorting (FACS) buffer (3% bovine serum albumin and 0.1% sodium azide in Dulbecco’s PBS) for 2 h at 4 °C. The cells were subsequently washed with FACS buffer and incubated at 4 °C for 1 h with 100 µL of antimouse IgG, IgG1, IgG2a, IgG2b, and IgG3 antibodies (Jackson ImmunoResearch) or with other indicated antibodies in Dulbecco’s PBS. After three additional washes, the cells were acquired and analyzed using a BD FACSVerse flow cytometer. Data were processed using FlowJo version 10.7 (BD, Ashland, OR, USA).

### Immunoblotting and immunoprecipitation

Cells were lysed in ice-cold lysis buffer (50 mM Tris-HCl, pH 7.5, 150 mM NaCl, 2 mM ethylenediaminetetraacetic acid, 1% Triton X-100, and 0.5% NP-40) supplemented with a protease and phosphatase inhibitor cocktail (Roche, Basel, Switzerland) for 30 min. The lysates were then homogenized through sonication on ice using a Branson Ultrasonics Sonifier 250 in three 10-s bursts separated by 30-s intervals. Following sonication, the samples were incubated on ice for 30 min. Cellular extracts were collected by centrifugation at 12,000 × *g* for 30 min, and protein concentrations were determined using a Bio-Rad Protein Assay (Bio-Rad).

For immunoprecipitation, cells were first cross-linked with dithiobis(succinimidyl propionate) (DSP) (Thermo Fisher Scientific, Cleveland, OH, USA) for 30 min and lysed in lysis buffer. Cellular extracts (500 μg) were incubated with the indicated primary antibody overnight at 4 °C, followed by a 2-h incubation with Protein G Dynabeads magnetic beads (#10003D, Thermo Fisher Scientific, Cleveland, OH, USA). The immunocomplexes were pelleted through centrifugation, washed thrice with lysis buffer, and resuspended in sodium dodecyl sulfate–polyacrylamide gel electrophoresis (SDS-PAGE) sample-loading buffer (50 mM Tris-HCl, pH 6.8, 10% glycerol, 2% sodium dodecyl phosphate, 20 mM β-mercaptoethanol, and 0.1% bromophenol blue). The immunocomplexes were separated using SDS-PAGE and transferred onto polyvinylidene fluoride membranes (Millipore). The membranes were blocked with 10% skim milk in tris-buffered saline with Tween 20 (TBST; 50 mM Tris-HCl, pH 7.6, 150 mM NaCl, 0.05% Tween-20) for 1 h at room temperature. The membranes were subsequently incubated overnight at 4 °C with the indicated primary antibody (Munc18-2, #66238-1, Proteintech; Syntaxin-3, #15556-1, Proteintech; FasL, #BS-0216R, Bioss; β-actin, MAB1501, Millipore), followed by incubation with horseradish peroxidase–conjugated secondary antibody (Jackson ImmunoResearch) for 1 h at room temperature. Immunoreactive signals were detected using Luminata Western Chemiluminescent horseradish peroxidase substrates (Millipore), per the manufacturer’s protocol. Data were excluded from analysis only if technical errors or sample loss were documented prospectively; no arbitrary exclusions were made.

### Immunofluorescence

Cells were seeded on coverslips and cultured overnight. The following day, the cells were treated and fixed in methanol at −20 °C for 3–7 min. After fixation, the cells were blocked with 1% BSA in PBST at room temperature (25 °C) for 1 h. Coverslips were incubated with the respective primary antibodies (Munc18-2, #66238-1, Proteintech; Syntaxin-3, #15556-1, Proteintech; FasL, #106603, Biolegend; LAMP1, #121602, Biolegend) overnight at 4 °C. After being washed with PBS, the cells were stained with a fluorescent-conjugated secondary antibody (Jackson ImmunoResearch) at room temperature for 1 h. Following extensive washes in PBS, the coverslips were mounted using 4′,6-diamidino-2-phenylin-dole DAPI Fluoromount-G (#0100-20, SouthernBiotech) to counterstain the cell nuclei. Images were captured using a Zeiss LSM 880 confocal microscope (Zeiss) with a 60× objective lens. For tissue immunofluorescence, the spleens were fixed in 10% formalin, sectioned, and prepared for staining. The sections were blocked with 0.3% BSA in 0.1% Triton X-100 at room temperature for 1 h and incubated overnight at 4 °C with primary antibodies: Ki67 (1:300, ARG53222, Arigo), IL21 (1:100, 17625-1, Proteintech), CD3 (1:200, GB12014, Servicebio), and B220 (1:50, 103228, Biolegend). The sections were incubated with the corresponding fluorescent-conjugated secondary antibodies (1:200, Jackson ImmunoResearch) for 1 h at 37 °C. The tissue slides were scanned using an Olympus VS200 scanner.

### Electroporation and small interfering ribonucleic acid (siRNA) for gene targeting

siRNAs targeting Munc18-2 (Stxbp2, assay IDs s74554 and s74555), Syntaxin-3 (Stx3, assay IDs s74545 and s74547) and control siRNAs were purchased from Ambion (Silencer Negative Control siRNA; AM4611; Waltham, MA, USA). siRNA delivery was conducted using a Neon Transfection System 10 µL kit (Thermo Fisher, Catalog #MPK1025) following the manufacturer’s protocol. For each condition, 2 × 10^5^ cells were centrifuged at 300 × *g* for 5 min, washed with PBS, centrifuged again, and resuspended in R buffer. Electroporation was performed using 10 µL Neon tips, which were used a maximum of two times as technical replicates. Each electroporation consisted of three pulses at 1400 V, each lasting 20 ms. Following electroporation, the cells were immediately transferred to a recovery buffer.

### Activation-induced cell death assay

CD4⁺ T cells were isolated from the spleens and lymph nodes of BALB/c mice using anti-mouse CD4 T cell MicroBeads (MACS). The purity of the isolated CD4⁺ T cell population was greater than 85%. Cells were activated for 48 h with Dynabeads CD3/CD28 (Gibco) per the manufacturer’s instructions. After activation, the CD4⁺ T cells were labeled with CellTrace CFSE dye (Thermo Fisher Scientific). Macrophages activated with LPS at the indicated concentrations were cocultured with the activated CD4⁺ T cells at a ratio of either 1:1 or 5:1. Cell death was assessed through staining with PI and annexin V.

### Panoramic spleen immunofluorescence imaging and QuPath-based cell mapping

Spleens were fixed in 10% neutral buffered formalin, processed for paraffin embedding, and sectioned at 3–5 μm. Sections were immunostained for CD4, CD8, and CD49b (NK cells), with DAPI used to label nuclei. Whole-section images were acquired on a Leica microscope at 40× using identical acquisition settings across groups, and tiled fields were stitched to generate panoramic spleen composites. For analysis, the splenic tissue area was manually annotated in QuPath, including the entire spleen section while excluding obvious staining artifacts and large blood vessels. Nuclei were segmented from the DAPI channel using the Cellpose extension in QuPath; detected nuclei were expanded by a fixed distance (expansion) to approximate per-cell measurement regions. Mean fluorescence intensities for CD4, CD8, and CD49b were measured per expanded cell ROI, and cells were classified using predefined intensity thresholds applied uniformly across all samples as CD4⁺, CD8⁺, CD4⁺/CD8⁺ double-positive, CD49b⁺ NK, or other. Cell density was calculated as the number of classified cells divided by the annotated tissue area (cells/mm²), and subset frequency was calculated as the percentage of each class among all DAPI⁺ nucleated cells. Each symbol represents one mouse (one analyzed section per mouse). P values shown in Supplementary Fig. S2 correspond to a single pre-specified comparison between genotypes under allosensitization (*Znrf1*^f/f^ vs *Znrf1*^myeΔ^) using one-way ANOVA; no other pairwise comparisons were performed.

### Colocalization analysis

Confocal z-stacks were acquired with identical settings across conditions and converted to maximum-intensity projections after uniform background subtraction and 16-bit conversion. For each cell, an outer whole-cell contour (including lamellipodia/filopodia; cell–cell contacts/debris excluded) was drawn and a 1-µm inner inset generated; the cortical ring ROI was the 0–1 µm annulus between these contours, and the whole-cell ROI corresponded to the outer contour. Intensity-based colocalization was computed in FIJI/ImageJ (Coloc2) with Costes automatic thresholding (Channel 1 = FasL-488; Channel 2 = LAMP1-594), reporting Manders’ tM1 (fraction of FasL overlapping LAMP1) separately in cortex and whole-cell. Cortical enrichment was defined as ΔtM1 = tM1(cortex)—tM1(whole-cell). QC: only ROIs with Costes *P* ≥ 0.90 and non-degenerate thresholds were accepted; ΔtM1 was calculated only when both ROIs passed QC.

Object-based triple colocalization of FasL (Ch1), Syntaxin-3/Stx3 (Ch2), and Munc18-2 (Ch3) was performed using ColocQuant/ColocJ (FIJI/ImageJ; protocol in Supplementary File). Puncta were detected on projections with a LoG spot-enhancing filter and channel-specific thresholds (mean + c·SD); mutual nearest-neighbor assignment within Dmax = 2 px defined two- and three-channel colocalization (LoG σ: Ch1 = 1.4 px, Ch2 = 1.4 px, Ch3 = 1.8 px; thresholds: Ch1 = 2.1, Ch2 = 2.3, Ch3 = 2.3). Readouts included per-cell counts (triple and pairs) and perimeter-normalized densities (puncta/µm) using the same whole-cell contour. Statistics: normality/equal-variance (Shapiro–Wilk; Brown–Forsythe), then Kruskal–Wallis with Dunn’s post-hoc for prespecified contrasts; Wilcoxon signed-rank vs 0 for ΔtM1.

### Data analysis

Group sizes were selected based on prior experience and published studies for the corresponding assays, while minimizing animal use. The exact n for each experiment/condition is stated in the figure legends, and n represents biological replicates (individual mice or independent cell preparations) unless otherwise noted. Data were analyzed for statistical significance using SigmaPlot (Systat Software). Results are expressed as mean ± standard deviation(SD). For comparisons between two groups, statistical significance was determined using an unpaired, two-tailed Student’s *t* test or Welch’s t test, as appropriate based on variance equality. A one-way analysis of variance (ANOVA) was used for comparisons involving more than two groups, followed by Dunnett’s multiple comparisons test. A two-way ANOVA was conducted for comparisons of more than two groups across multiple time points. Homogeneity of variance was evaluated prior to between-group comparisons; when variances were unequal, appropriate analyses that do not assume equal variance were applied. A *p* value of <0.05 was considered statistically significant. No animals or data were excluded from analysis unless a clear technical error (e.g., sample loss or improper processing) was documented prospectively. Investigators were not blinded to group allocation during the experiments and outcome assessment.

## Results

### ZNRF1 deficiency in myeloid cells drives spontaneous splenic hyperactivation and primes humoral responses

**Fig. 1:**
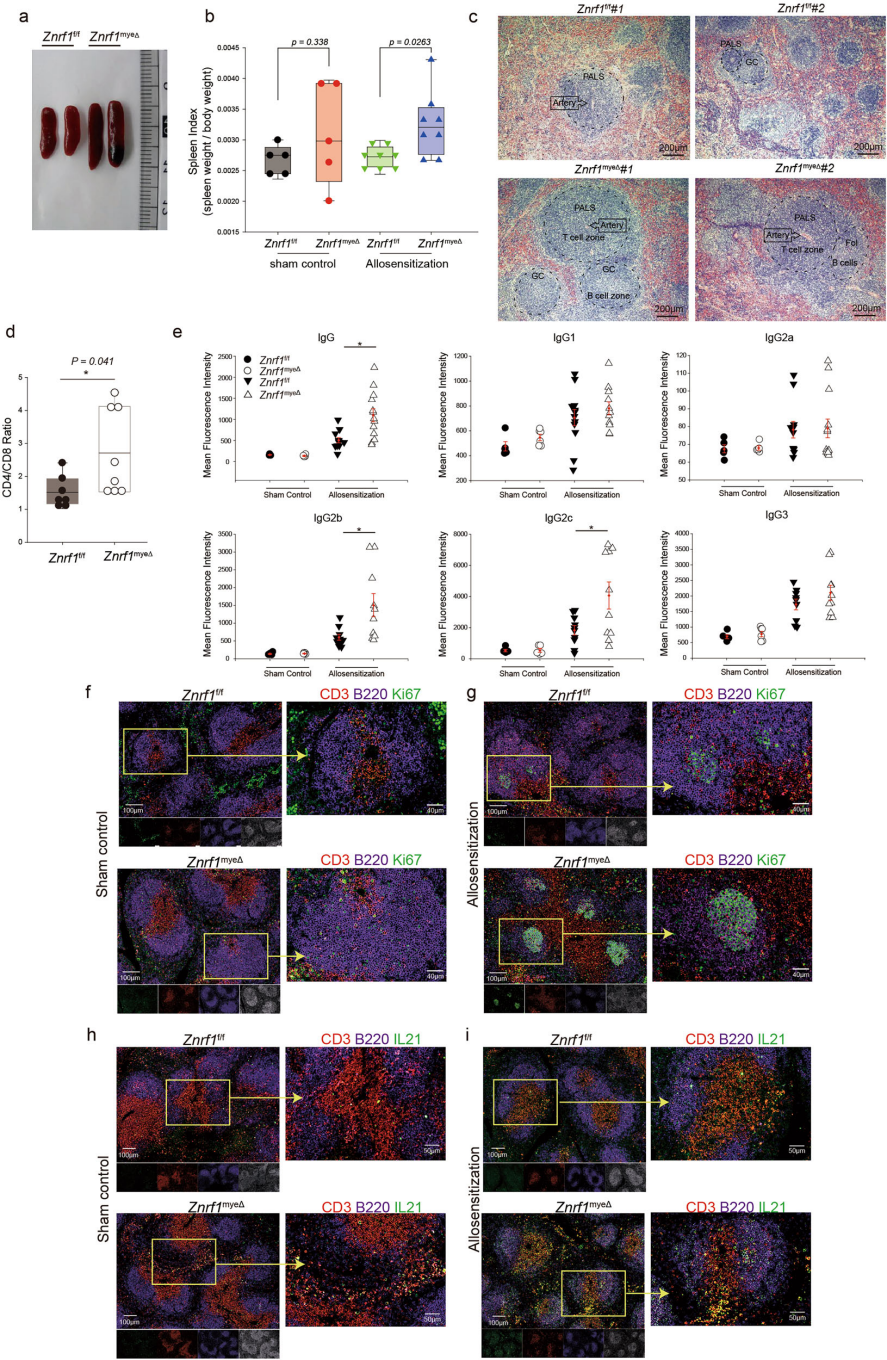
ZNRF1 loss in myeloid cells drives age-dependent splenic hyperactivation and enhances allosensitization-induced humoral responses. **a**, **c** show the spontaneous phenotype in aged mice (24–32 weeks), whereas **b**,**d**–**i** show analyses in 6–8-week-old mice under sham or blood-transfusion allosensitization. In the allosensitization model, sera were collected at day 14 post-transfusion, while spleens were collected at day 10 to assess earlier tissue remodeling/GC reactions. **a** Representative spleens from aged *Znrf1*^f/f^ and *Znrf1*^myeΔ^ littermates, showing spontaneous spleen enlargement in *Znrf1*^myeΔ^ mice. **b** Spleen index (spleen weight/body weight) in 6–8-week-old *Znrf1*^f/f^ and *Znrf1*^myeΔ^ mice under sham control conditions (*n* = 5 per genotype) or 14 days after allosensitization (*n* = 8 per genotype). Welch’s t-test (two-tailed); *p* values are shown. **c** H&E staining of spleen sections from aged (24–32 weeks) *Znrf1*^f/f^ and *Znrf1*^myeΔ^ littermates, highlighting the periarteriolar lymphoid sheath (PALS), T-cell zones, B-cell zones, and germinal centers (GCs). Scale bars, 200 µm. **d** Flow cytometry analysis of the splenic CD4/CD8 T-cell ratio in 6–8-week-old mice at day 14 after allosensitization (*Znrf1*^f/f^, *n* = 7; *Znrf1*^myeΔ^, *n* = 8). **e** Serum alloantibody levels measured by flow cytometry 14 days after allosensitization, including total IgG and IgG subclasses (IgG1, IgG2a, IgG2b, IgG2c, and IgG3). Sera were obtained from two independent allosensitization batches, and all samples were stained and acquired in the same flow cytometry run to minimize inter-run variability. Each symbol represents one mouse. Sample sizes (*Znrf1*^f/f^ sham, *Znrf1*^myeΔ^ sham, *Znrf1*^f/f^ allo, *Znrf1*^myeΔ^ allo) were: IgG (*n* = 4, 4, 12, 13) [4, 12, 13]; IgG1 (*n* = 5, 6, 12, 11) [5, 6, 11, 12]; IgG2a (*n* = 5, 5, 11, 12) [5, 11, 12]; IgG2b (*n* = 5, 6, 10, 10) [5, 6, 10]; IgG2c (*n* = 5, 5, 10, 10) [5, 10]; IgG3 (*n* = 5, 6, 9, 10) [5, 6, 9, 10]. Differences in n reflect serum availability and/or assay QC for individual subclasses. **f**, **g** Representative immunofluorescence images of spleens from sham-treated mice or mice collected 10 days after allosensitization. Sections were stained for Ki67 (green), CD3 (red), and B220 (purple). **h**, **i** Sections were stained for IL-21 (green), CD3 (red), and B220 (purple).

The spleen is a key secondary lymphoid organ orchestrating both innate and adaptive immune responses, with its architecture and cellular composition tightly regulated under homeostasis. Disruption of splenic lymphoid tissue homeostasis can result in splenomegaly, reflecting lymphoproliferative or immune-activated states [15]. To dissect the role of ZNRF1 in myeloid cells in immune regulation, we generated myeloid-specific *Znrf1* knockout mice (*Znrf1*^myeΔ^) on a C57BL/6 background and compared them with *Znrf1*^f/f^ littermate controls. In 6–8-week-old mice under sham conditions, spleen index (spleen weight/body weight) was comparable between genotypes (Fig. 1b, Welch’s t-test, *p* = 0.338), with no overt difference in gross spleen appearance in the baseline cohort (Supplementary Fig. S1). In contrast, aged *Znrf1*^myeΔ^ mice (24–32 weeks) spontaneously developed splenomegaly (Fig. 1a). Histological assessment provided further insight into splenic architecture. Examination of spleen sections revealed substantial enlargement of germinal centers (GCs) within the white pulp in *Znrf1*^myeΔ^ mice compared with *Znrf1*^f/f^ controls (Fig. 1c). In addition, the periarteriolar lymphoid sheath (PALS), a region predominantly comprised of T cells, was expanded in *Znrf1*^myeΔ^ spleens, while the overall demarcation between GC, T-cell, and B-cell compartments remained generally preserved, suggesting hyperplasia rather than overt disorganization. Collectively, these findings indicate that deletion of ZNRF1 in myeloid cells is sufficient to drive spontaneous splenic lymphoid hyperplasia with prominent GC expansion.

### Influence of Znrf1 deficiency on adaptive immune response

We next asked whether myeloid ZNRF1 deficiency augments antigen-driven humoral immunity using a blood-transfusion allosensitization model in which *Znrf1*^f/f^ and *Znrf1*^myeΔ^ mice were transfused with whole blood from BALB/c donors. Sera were collected at day 14 post-transfusion to quantify alloantibodies, whereas spleens were collected at day 10 to capture earlier tissue remodeling and GC reactions. At day 14, allosensitized *Znrf1*^myeΔ^ mice exhibited a higher spleen index than *Znrf1*^f/f^ controls (Fig. 1b, Welch’s t-test, *p* = 0.0263). Flow cytometric analysis of splenic T-cell subsets at day 14 showed a significant increase in the CD4/CD8 T-cell ratio in *Znrf1*^myeΔ^ mice (Fig. 1d, *p* = 0.041), indicating a shift in T-cell subset balance following alloantigen exposure. Consistent with enhanced humoral activation, allosensitized *Znrf1*^myeΔ^ mice developed higher levels of class-switched alloantibodies, with significant increases in total IgG as well as IgG2b and IgG2c compared with *Znrf1*^f/f^ controls and sham-treated mice (Fig. 1e). Increases in IgG1, IgG2a, and IgG3 were less prominent, indicating selective enhancement of specific class-switch pathways in the context of myeloid ZNRF1 loss. These data suggest that myeloid ZNRF1 plays a negative regulatory role in humoral alloimmunity. To determine whether the elevated CD4/CD8 ratio reflected changes in the abundance of individual lymphocyte subsets and to assess NK cells in an orthogonal, tissue-wide manner, we performed panoramic whole-spleen immunofluorescence staining for CD4, CD8, and CD49b (NK cells), followed by image-based cell mapping and quantification across entire spleen sections (Supplementary Fig. S2). In sham-treated mice, CD4⁺ and CD8⁺ T-cell densities (cells/mm²) and their frequencies among DAPI⁺ nucleated cells were comparable between genotypes. Following allosensitization, however, *Znrf1*^myeΔ^ spleens showed an increased density/frequency of CD4⁺ T cells together with a reduced density/frequency of CD8⁺ T cells relative to *Znrf1*^f/f^ controls, consistent with the increased CD4/CD8 ratio observed by flow cytometry. NK cells remained a minor population and did not show a consistent genotype-dependent change (Supplementary Fig. S2).

To link the augmented alloantibody response to GC activity, we performed immunofluorescence staining for Ki67, a marker of cellular proliferation, in spleen sections collected 10 days after allosensitization. Under sham control conditions, both genotypes exhibited rare Ki67⁺ B cells within the germinal centers (Fig. 1f). However, following allosensitization, *Znrf1*^myeΔ^ spleens displayed obvious expansion of germinal centers accompanied by increased numbers of Ki67⁺ B cells, indicative of increased B-cell clonal proliferation and ongoing GC activity (Fig. 1g). This enhanced proliferative response in *Znrf1*^myeΔ^ mice is in line with their increased capacity to generate alloantigen-specific antibodies. We next examined the role of follicular helper T (Tfh) cells and their cytokine IL-21, which is known to drive B cell differentiation, class switching, and plasma cell generation [16–19]. Immunofluorescence analysis revealed that, under sham control conditions, both *Znrf1*^f/f^ and *Znrf1*^myeΔ^ mice showed minimal IL-21 expression within CD3⁺ T cell zones or B cell follicles (Fig. 1h). In contrast, after alloantigen stimulation, IL-21 expression was markedly upregulated in both CD3⁺ T cell areas and B220⁺ B cell regions, with the increase being more pronounced in *Znrf1*^myeΔ^ mice (Fig. 1i). Furthermore, IL-21⁺ B cells were more frequently observed in follicles of *Znrf1*^myeΔ^ mice, suggesting that myeloid ZNRF1 deficiency fosters a microenvironment that promotes Tfh-mediated B cell help and facilitates plasma cell differentiation [20, 21]. Taken together, these data reveal that ZNRF1 deficiency in myeloid cells promotes spontaneous splenic lymphoid hyperplasia and primes the adaptive immune system for exaggerated humoral responses upon alloantigen exposure. Mechanistically, this phenotype is associated with expanded germinal center reactions, increased Tfh cell and IL-21 activity, and preferential production of class-switched alloantibodies [16–21]. These in vivo changes prompted us to test whether a macrophage-intrinsic checkpoint—namely the FasL pathway that contracts activated T-cell responses—becomes impaired when ZNRF1 is lost [22–24].

### ZNRF1 regulates surface expression of FasL

**Fig. 2:**
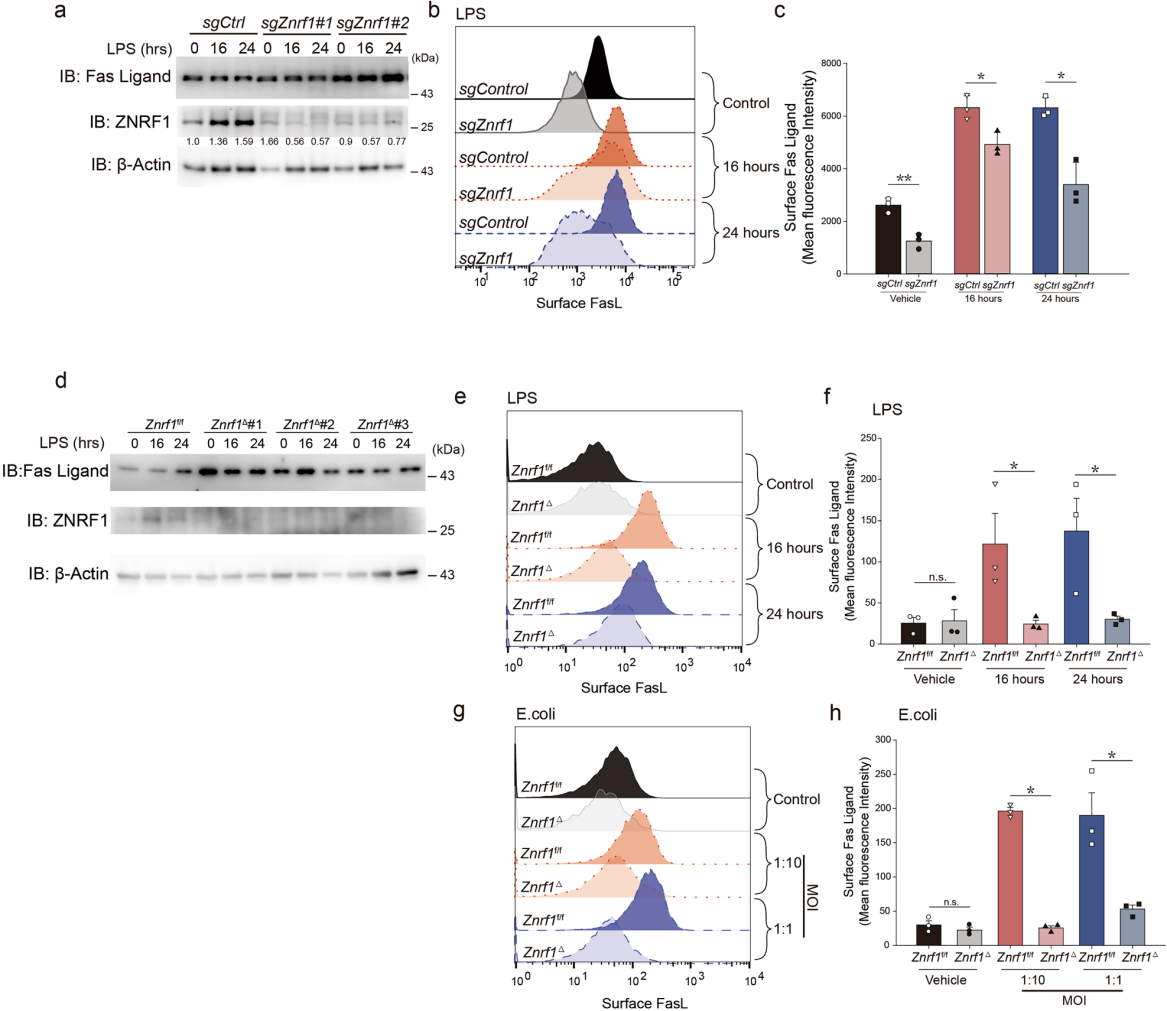
ZNRF1 regulates surface Fas ligand expression in macrophages. **a** Immunoblot analysis of FasL and ZNRF1 in control (*sgCtrl*) and *Znrf1*-targeted RAW264.7 macrophage clones (*sgZnrf1* #1 and #2) stimulated with lipopolysaccharide (LPS, 100 ng/mL) for the indicated times (0, 16, and 24 h). β-Actin served as a loading control. Numbers beneath the ZNRF1 blot indicate densitometric quantification after lane-specific background subtraction (ZNRF1/β-actin), normalized to *sgCtrl* 0 h (= 1). **b** Representative flow-cytometry histograms of surface FasL in *sgCtrl* and *sgZnrf1* macrophages under vehicle control conditions or after LPS stimulation for 16 or 24 h. **c** Quantification of surface FasL mean fluorescence intensity (MFI) from (**b**) (*n* = 3 biological replicates per group; representative of three independent experiments). **d** Immunoblot analysis of FasL and ZNRF1 in bone-marrow-derived macrophages (BMDMs) from *Znrf1*^f/f^ and *Znrf1*^Δ^mice stimulated with LPS (100 ng/mL) for the indicated times. **e** Representative flow-cytometry histograms of surface FasL in *Znrf1*^f/f^ and *Znrf1*^Δ^ BMDMs following LPS stimulation. **f** Quantification of surface FasL MFI from (**e**) (*n* = 3 biological replicates per group; representative of two independent experiments). **g** Representative flow-cytometry histograms of surface FasL in *Znrf1*^f/f^ and *Znrf1*^Δ^ BMDMs stimulated with live *E. coli* at the indicated multiplicities of infection (MOI). **h** Quantification of surface FasL MFI from (**g**) (*n* = 3 biological replicates per group; representative of two independent experiments). Data are shown as mean ± SD. * *p* < 0.05, ** *p* < 0.01; n.s., not significant (unpaired two-tailed t-test).

Proper regulation of Fas ligand (FasL) surface expression is critical for maintaining immune homeostasis, as evidenced by the development of splenomegaly, lymphadenopathy, B cell hyperactivation, and autoimmunity in FasL-deficient (*Faslg*^−/−^) mice [22, 23]. FasL is primarily regulated at the post-translational level through trafficking from intracellular stores to the plasma membrane, rather than by changes in total protein abundance [25, 26]. This raises the question of whether ZNRF1 controls the surface delivery of FasL, a mechanism potentially underlying the hyperactive immune phenotype observed in *Znrf1*-deficient myeloid cells. To assess this, we generated two independent CRISPR-edited *Znrf1*-deficient RAW264.7 macrophage clones (*sgZnrf1* #1 and #2), with non-targeting controls (*sgCtrl*) as comparison. Because ZNRF1 is inducible by LPS, its basal signal at 0 h is near background; therefore, we quantified ZNRF1 immunoblot signals by grayscale densitometry (normalized to β-actin) and report the values beneath the corresponding lanes (Fig. 2a), confirming markedly attenuated/absent LPS-induced ZNRF1 accumulation in both *sgZnrf1* clones. Immunoblot analysis revealed that, under resting conditions, total FasL protein levels were similar between *sgCtrl* and *sgZnrf1* macrophages. Upon LPS stimulation, however, total FasL protein levels in *sgZnrf1* clones increased more than in controls, indicating that ZNRF1 is not required for the induction or synthesis of FasL protein (Fig. 2a). In contrast, surface FasL expression revealed a strikingly different pattern. In *sgCtrl* macrophages, LPS stimulation for 16–24 h induced a marked increase in surface FasL, as shown by a rightward shift in fluorescence histograms and significantly elevated mean fluorescence intensity (Figs. 2b, c; *p* < 0.05 as indicated). However, in *sgZnrf1* cells, surface FasL expression was lower at baseline and failed to increase in response to LPS, despite total cellular FasL being elevated. This dissociation indicates that ZNRF1 is required for the efficient trafficking and presentation of FasL at the plasma membrane upon activation, rather than its biosynthesis.

To confirm these findings in primary cells, bone-marrow-derived macrophages (BMDMs) were prepared from *Znrf1*^f/f^ and inducible knockout Mx1-Cre:*Znrf1*^Δ^ mice. Upon LPS stimulation, immunoblotting again revealed increased total FasL protein in *Znrf1*^Δ^ BMDMs, paralleling results in RAW264.7 cells (Fig. 2d). However, quantification of surface FasL by flow cytometry demonstrated that, whereas *Znrf1*^f/f^ BMDMs showed marked induction of surface FasL upon LPS stimulation, *Znrf1*^Δ^ BMDMs failed to mount a comparable response (Fig. 2e, f). At both 16 and 24 h post-LPS, surface FasL expression was significantly lower in *Znrf1*^Δ^ BMDMs than in controls (*P* < 0.05 or as shown in the figure). These data further support a model in which ZNRF1 is essential for the activation-induced mobilization of FasL to the cell surface. We also challenged BMDMs with *E. coli* (MOI 1:10 and 1:1); *Znrf1*^f/f^ cells mounted strong surface-FasL increases, whereas *Znrf1*^Δ^ cells remained hyporesponsive (Fig. 2g, h; *P* < 0.05). These primary-cell data corroborate that ZNRF1 is required for TLR- or pathogen-induced delivery of FasL to the plasma membrane. To test whether the impaired surface FasL reflects a selective trafficking defect rather than a broad alteration of activation-induced surface programs, we examined two control surface markers: CD95 (Fas), the cognate FasL receptor, and PD-L1, an independent immune checkpoint marker. The upregulation of the Fas receptor (CD95) upon stimulation was not significantly different between sgCtrl and *sgZnrf1* cells (Supplementary Fig. S3a). Similarly, analysis of programmed death ligand 1 (PD-L1), a well-established immune checkpoint molecule involved in T cell regulation [27]. revealed no significant changes in its expression under either basal or LPS-stimulated conditions in the absence of ZNRF1 (Supplementary Fig. S3b). Coupled with the divergence between total and surface FasL [25, 26], these findings indicate that ZNRF1 may operate at a post-translational trafficking/docking step that licenses stimulus-induced FasL exposure at the macrophage surface.

### ZNRF1 deficiency disrupts munc18-2 and syntaxin 3 interaction, leading to mislocalization of FasL in macrophages

FasL is trafficked in immune and endothelial cells within lysosome-related organelles (LROs) and is displayed at the plasma membrane upon activation [5, 26, 28]. LAMP1 marks these LROs and partially colocalizes with FasL during transit [1]. To assess whether ZNRF1 influences this pathway, we imaged FasL and LAMP1 by confocal microscopy and quantified intensity-based colocalization using Costes-thresholded Manders’ tM1 (the fraction of FasL overlapping LAMP1) in two regions of interest: a cortex defined as the outer whole-cell contour and its 1 µm inner inset (0–1 µm annulus) and the whole-cell ROI (Supplementary Fig. S4). In resting (vehicle-treated) macrophages, both *sgCtrl* and *sgZnrf1* cells exhibited numerous small LAMP1⁺ puncta distributed throughout the cytoplasm, with intermittent overlap with FasL—consistent with basal storage in LROs (Supplementary Fig. S4a). Upon LPS stimulation for 20 h, *sgCtrl* cells underwent a striking reorganization: LAMP1⁺ vesicles formed a prominent annular cortical ring at the cell periphery, where they co-accumulated with FasL. This cortical annulus is evident as a continuous band of LAMP1⁺ and FasL⁺ puncta adjacent to the plasma membrane, and line-scan profiles showed multiple coincident peaks for both markers, consistent with vesicle docking or retention at peripheral sites (Supplementary Fig. S4b, top). In contrast, *sgZnrf1* cells also developed a peripheral LAMP1⁺ annulus after LPS; however, FasL did not co-accumulate at the cortex and instead remained largely perinuclear, yielding sparse or offset peaks in the signal-intensity profiles (Supplementary Fig. S4b, bottom). To quantify these differences, we measured Costes-thresholded Manders’ tM1 within the cortex (0–1 µm annulus) and the whole-cell ROIs (Supplementary Fig. S4c). Cortical tM1 was lower in LPS-treated *sgZnrf1* than in LPS-treated *sgCtrl* macrophages (*p* = 0.006), and LPS increased cortical tM1 within *sgCtrl* (vehicle- vs LPS-treated, *p* = 0.013), whereas whole-cell tM1 did not differ among groups (all *p* > 0.05) (Supplementary Fig. S4d–e). Because cortical Manders’ tM1 can be influenced by cell-to-cell variability in global colocalization, we further computed a per-cell enrichment metric, ΔtM1 = tM1(cortex)—tM1(whole-cell) for each cell. ΔtM1 > 0 denotes cortical enrichment beyond the whole-cell baseline, whereas ΔtM1 ≤ 0 indicates absent or negative enrichment. ΔtM1 was positive in LPS-treated *sgCtrl* but near-zero/negative in LPS-treated *sgZnrf1*, yielding higher ΔtM1 in LPS-treated *sgCtrl* vs LPS-treated *sgZnrf1* (*p* = 0.006) and an LPS effect within *sgCtrl* (*p* = 0.045) (Supplementary Fig. S4f). Thus, ZNRF1 is not required for LAMP1⁺ vesicle polarization per se, but is required to couple FasL cargo to LAMP1-defined LROs and deliver it to the cortical docking domain.

**Fig. 3:**
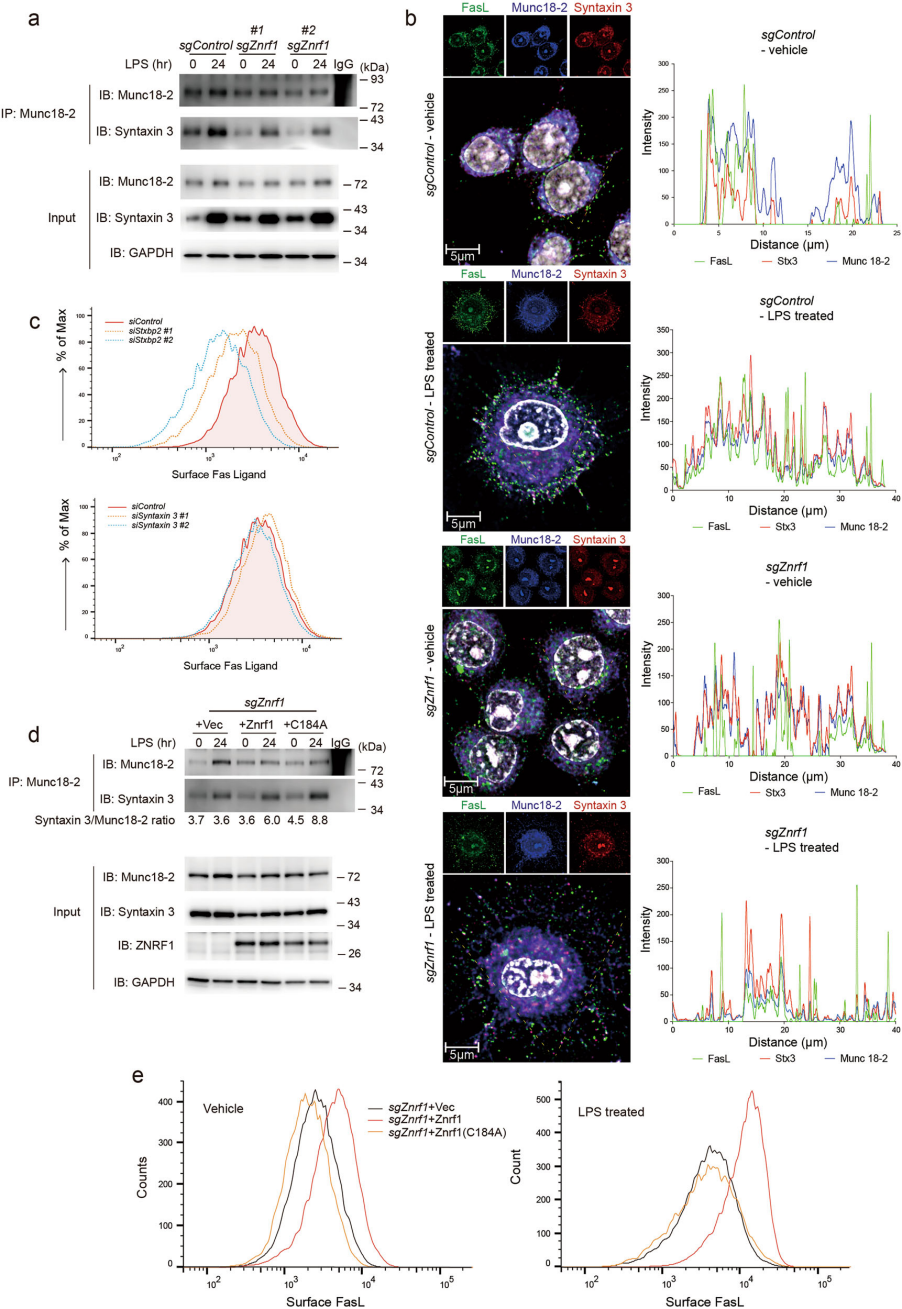
*ZNRF1* deficiency impaired interaction between Munc18-2 and Syntaxin 3. **a** Co-immunoprecipitation of Munc18-2 and Syntaxin-3 in control (*sgControl*) and *Znrf1*-deficient (*sgZnrf1*, two sgRNAs) murine macrophages after LPS (100 ng/mL, 24 h) stimulation. Cell lysates were immunoprecipitated with anti-Munc18-2 or IgG (control), followed by immunoblot analysis with specified antibodies. **b** Confocal microscopy of murine macrophages stained for FasL (green), Munc18-2 (blue), and Syntaxin-3 (red) under vehicle or LPS (20 h) conditions. Right panels: Signal profiles along the indicated dashed lines in each cell, showing fluorescence intensity for FasL (green), Munc18-2 (blue), and Syntaxin 3 (red). Scale bars, 5 µm. **c** In RAW264.7 macrophages, knockdown of Munc18-2 (*Stxbp2*) and Stx3 (*Syntaxin 3*) was achieved using siRNA targeting two distinct sequences. Following this genetic manipulation, the cells were harvested and analyzed for surface expression of FasL by using flow cytometry. **d** *Znrf1*-deficient macrophages were reconstituted with doxycycline-inducible wild-type ZNRF1, ZNRF1(C184A) mutant, or vector control. Following LPS stimulation (100 ng/mL, 24 h), co-immunoprecipitation was performed with anti-Munc18-2 and immunoblotting for Syntaxin-3 and Munc18-2. Syntaxin-3/Munc18-2 binding ratios are indicated. **e** Surface FasL expression was analyzed by flow cytometry in reconstituted macrophages from (**d**) under vehicle and LPS-treated conditions.

The terminal exposure of LRO cargo at the plasma membrane is mediated by SNARE proteins and the Sec1/Munc18 family of fusion accessory factors, particularly the interaction between Munc18-2 and Syntaxin-3 (Stx3), which together facilitate vesicle fusion [1]. To determine whether ZNRF1 modulates this machinery, we performed co-immunoprecipitation of Munc18-2 from *sgCtrl* and *sgZnrf1* RAW264.7 macrophages. Under resting conditions, the amount of Stx3 co-immunoprecipitated with Munc18-2 was reduced in *sgZnrf1* cells compared to *sgCtrl*, despite comparable total protein input (Fig. 3a). Following 24 h of LPS stimulation, both Munc18-2 and Stx3 increased in whole-cell lysates, but Stx3 association with Munc18-2 remained markedly diminished in *sgZnrf1* compared to *sgCtrl* (Fig. 3a). These results indicate that ZNRF1 promotes the assembly and stability of the Munc18-2–Stx3 complex under both basal and activated conditions.

To visualize how this disruption affects FasL trafficking, we used confocal imaging of FasL, Munc18-2, and Stx3. In *sgCtrl* macrophages after LPS stimulation, discrete peripheral puncta were observed where FasL, Munc18-2, and Stx3 colocalized near the cell cortex, as indicated by coincident peaks in signal-intensity plots (Fig. 3b, top two rows). These cortical tri-colocalized puncta are consistent with sites of functional vesicle docking and fusion. In contrast, *sgZnrf1* macrophages displayed FasL puncta that remained predominantly perinuclear and failed to colocalize with Munc18-2 and Stx3 at the periphery. The line scans showed clear separation between the peaks of the three proteins, further confirming defective coupling and peripheral docking of FasL vesicles (Fig. 3b, bottom two rows). To quantify the spatial coupling of FasL with the vesicle-fusion machinery, we performed object-based triple-colocalization of FasL (Ch1), Stx3 (Ch2) and Munc18-2 (Ch3) within whole-cell ROIs (including filopodia) using ColocQuant (σ: 1.4(Ch1)/1.4(Ch2)/1.8(Ch3); thresholds: 2.1/2.3/2.3; Dmax = 2 px) (Supplementary Fig. S5a) [29]. Upon LPS stimulation, *sgCtrl* macrophages displayed a robust increase in tri-colocalized puncta per cell and in the perimeter-normalized density (puncta per μm of the same cell outline), whereas *sgZnrf1* cells showed a significant attenuation in both metrics (Supplementary Fig. S5b,f). Object-based analyses within whole-cell ROIs (including filopodia) further quantified two-color colocalization and perimeter-normalized densities. Both FasL–Stx3 and FasL–Munc18-2 readouts showed significantly fewer events in *sgZnrf1* than *sgCtrl* after LPS, and the Munc18-2–Stx3 pair showed the same directional trend (Supplementary Fig. S5c-e, g-i). These quantitative data align with the reduced Stx3/Munc18-2 association and the diminished surface FasL, supporting a model in which ZNRF1 promotes Munc18-2–Stx3 complex assembly that licenses peripheral docking of FasL-positive vesicles.

### Role of E3 ubiquitin ligase activity of ZNRF1 in regulating Munc18-2, Syntaxin 3, and FasL surface expression in macrophages

Munc18-2 and Stx3 are core components of the secretory-lysosome fusion machinery; genetic or pharmacologic perturbation of these factors impairs degranulation in hematopoietic cells [30–32]. To place FasL trafficking within this axis, we depleted *Stxbp2* (Munc18-2) or *Stx3* by siRNA in RAW264.7 macrophages and quantified surface FasL by flow cytometry. *Stxbp2* knockdown reproducibly reduced FasL at the cell surface, whereas *Stx3* knockdown produced slight or no decrease under our conditions (Fig. 3c). These data position Munc18-2 as the rate-limiting determinant of FasL delivery/retention at the plasma membrane. Because ZNRF1 is a RING-domain E3 ubiquitin ligase [9, 32], we next asked whether its catalytic activity is required for assembly of the Munc18-2–Stx3 complex. Using a reconstitution approach in *sgZnrf1* macrophages, expression of ZNRF1 (WT) or the catalytically inactive ZNRF1(C184A) increased Stx3 recovery in Munc18-2 immunoprecipitates after LPS compared with vector controls (Fig. 3d), indicating that ZNRF1 protein can scaffold or stabilize the complex independent of catalysis. However, only ZNRF1 (WT) restored surface FasL under basal conditions and further enhanced it upon LPS stimulation, whereas ZNRF1(C184A) failed to do so and overlapped with vector controls (Fig. 3e). Together, these findings indicate that structural ZNRF1 supports assembly of the Munc18-2–Stx3 complex, but E3 ligase activity is indispensable for productive trafficking that culminates in FasL exposure at the plasma membrane—most plausibly by enabling vesicle priming/tethering and fusion competence downstream of complex assembly [9, 30, 32].

### Effect of ZNRF1 on FasL-mediated apoptosis and macrophage-T cell dynamics

**Fig. 4:**
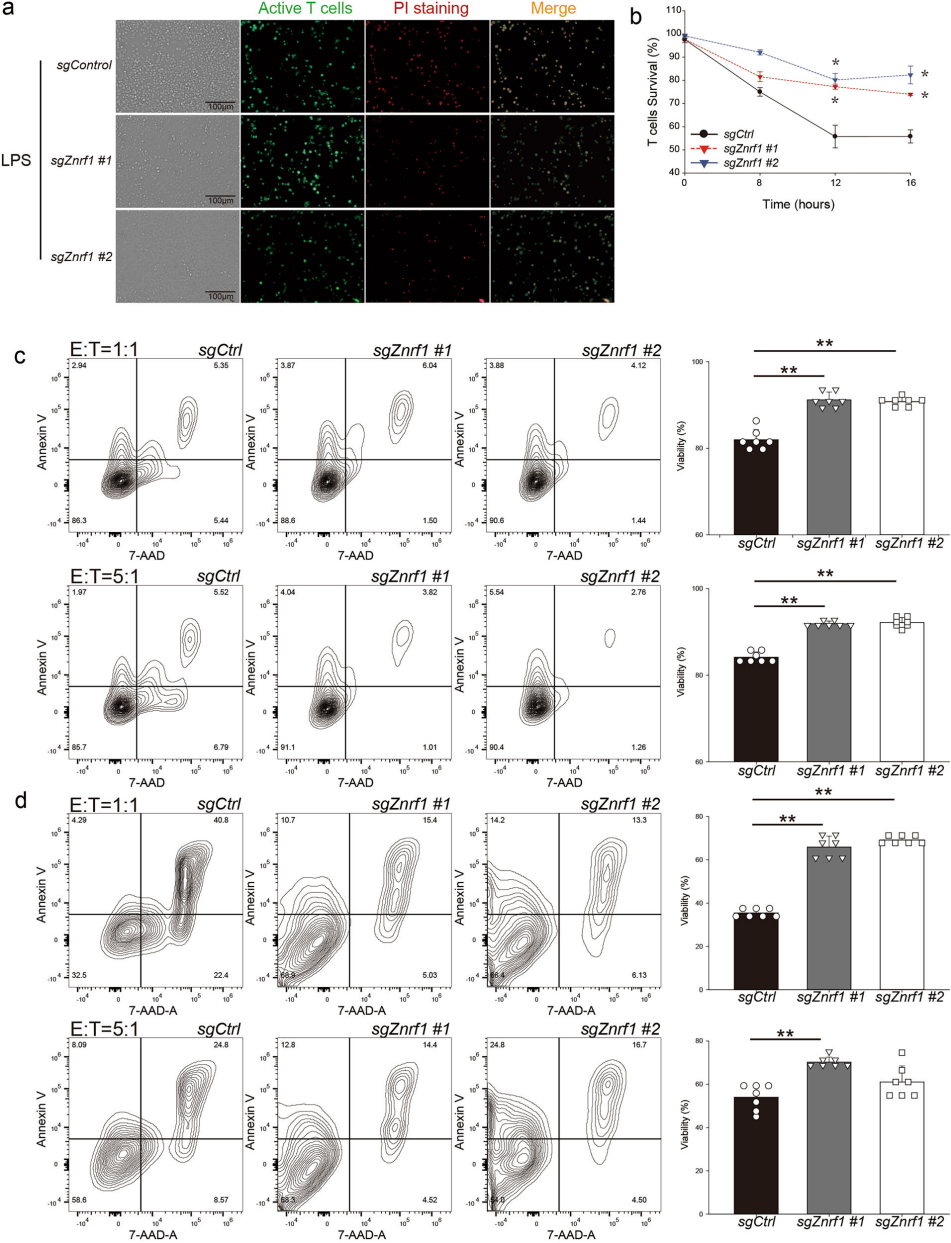
ZNRF1 promotes FasL-dependent cytotoxic activity of macrophages. **a** Wild-type (*sgControl*) and *Znrf1*-deficient (*sgZnrf1*) RAW264.7 macrophages were stimulated with lipopolysaccharide (LPS, 100 ng/mL) and co-cultured with carboxyfluorescein succinimidyl ester (CFSE)-labeled activated CD4⁺ T cells for 16 h. Propidium iodide (PI) was added to the medium to label dead cells. Representative images of CFSE (green), PI (red), and merged channels are shown. Scale bars, 100 μm. **b** T-cell death was quantified from time-lapse images by counting CFSE⁺PI⁺ cells; survival (%) was calculated as 100 - (% CFSE⁺PI⁺ among total CFSE⁺ cells). Data represent three independent experiments. **c** CFSE-labeled L1210-Fas cells were co-cultured with *sgControl* or *sgZnrf1* RAW264.7 macrophages at effector:target (E:T) ratios of 1:1 and 5:1 after LPS priming (100 ng/mL). Cell viability was assessed by flow cytometry using annexin V and 7-AAD staining. **d** CFSE-labeled L1210-Fas/F4/80 double-positive events were gated and analyzed by flow cytometry for annexin V and 7-AAD staining to assess cell death. Data are shown as mean ± SD from at least two independent experiments (*n* = 5–6 per group). **p < 0.01 (two-tailed t-test).

FasL-induced apoptosis is a major mechanism for contraction of activated T cells and termination of immune responses [24]. Our data show that *Znrf1* deletion leads to decreased surface FasL expression in macrophages, which suggests that *Znrf1*-deficient macrophages might have reduced ability to trigger apoptosis in activated T cells. To test the hypothesis, we performed live-cell imaging and flow cytometry-based cytotoxicity assays using LPS-primed RAW264.7 macrophages cocultured with activated CD4⁺ T cells. LPS-primed RAW264.7 macrophages were co-cultured with CFSE-labeled, CD3/CD28-activated CD4⁺ T cells; propidium iodide (PI) uptake was monitored for 16 h (Fig. 4a). T-cell survival curves showed that co-culture with *sgCtrl* macrophages resulted in progressive loss of viability (<60%), whereas *sgZnrf1* clones (#1 and #2) preserved substantially higher T-cell survival (~80% of T cells remained viable) (Fig. 4b). The survival gap was statistically significant after 12–16 h, supporting the conclusion that *Znrf1* deficiency in macrophages impairs their ability to kill T cells.

To complement the time-lapse imaging assay, we quantified target-cell death by endpoint flow cytometry using annexin V/7-AAD staining. The experiment involved co-culturing CFSE-labeled Fas-expressing L1210 targets (L1210-Fas) with LPS-primed *sgControl* or *sgZnrf1* RAW264.7 macrophages for 16 h at two effector-to-target (E:T) ratios of 1:1 and 5:1. Analysis of the total target-cell population (CFSE⁺) showed that *sgControl* macrophages induced greater target-cell death but both independent *sgZnrf1* clones consistently preserved higher target-cell viability across both E:T ratios (Fig. 4c). We further analyzed CFSE⁺F4/80⁺ events (macrophage-associated targets) to enrich for target cells physically associated with macrophages (e.g., through cell–cell association or engulfment); this analysis similarly showed reduced cytotoxic readouts in *sgZnrf1* macrophages (Fig. 4d). Under higher-dose LPS priming (500 ng/mL), *sgControl* macrophages further decreased L1210-Fas viability, whereas *sgZnrf1* macrophages maintained comparatively higher target-cell survival (Supplementary Fig. S6). The endpoint apoptosis measurements confirm the live-imaging assay results from Fig. 4a, b, supporting the conclusion that ZNRF1 enhances macrophage killing of Fas-expressing cells. These findings collectively indicate that ZNRF1-deficient macrophages exhibit reduced ability to induce Fas-mediated apoptosis in activated T cells and Fas-expressing targets, aligning with the previously documented impaired surface presentation of FasL and the recognized role of FasL in terminating T-cell responses [24]. The maintained target-cell viability in the presence of *sgZnrf1* effectors across stimulant concentrations and E:T ratios indicates a fundamental defect in FasL-mediated cytotoxic function.

## Discussion

**Fig. 5:**
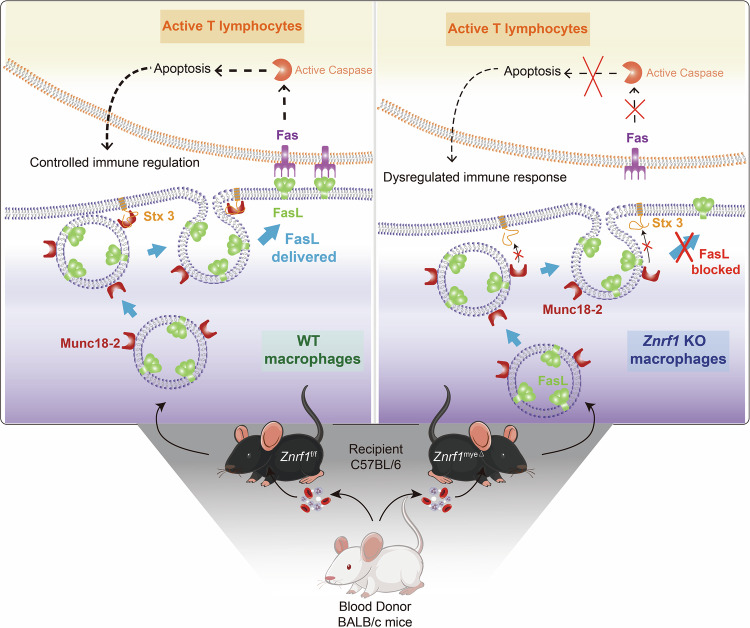
ZNRF1 regulates FasL vesicle trafficking and immune homeostasis. Schematic illustration of FasL trafficking and T cell apoptosis in wild-type (WT, left) versus *Znrf1*-deficient (right) macrophages. In WT macrophages, ZNRF1 promotes the interaction between Munc18-2 and Syntaxin-3 (Stx3), enabling efficient delivery of FasL-containing vesicles to the plasma membrane and triggering FasL-mediated apoptosis of activated T lymphocytes. In *Znrf1*-deficient macrophages, disruption of the Munc18-2/Syntaxin-3 interaction impairs FasL vesicle trafficking and surface expression, resulting in defective Fas-mediated T cell apoptosis and immune dysregulation. The lower panel depicts the in vivo allogeneic transfusion model, in which C57BL/6 *Znrf1*^f/f^ or *Znrf1*^myeΔ^ recipients received BALB/c whole blood via intravenous injection.

Immune homeostasis depends not only on ligand–receptor expression but also on the spatiotemporal control of membrane trafficking that determines when and where effectors appear at the cell surface. In myeloid cells, these post-translational checkpoints remain comparatively underexplored. ZNRF1—a RING-type E3 ubiquitin ligase previously implicated in innate immune regulation, receptor turnover, TLR routing, and IL-10 production [11, 33]—emerges here as a gatekeeper of stimulus-induced Fas ligand (FasL) exposure in macrophages. Our findings reveal that in wild-type macrophages (Fig. 5), activation drives FasL-bearing lysosome-related organelles (LROs) to the cortex, where a Munc18-2–Stx3 fusion module—enabled by ZNRF1—mediates docking/fusion and surface exposure of FasL. Surface FasL engages Fas on neighboring activated T lymphocytes, triggering caspase activation and apoptosis to contract the response [24–26, 30–32]. In *Znrf1-*deficient macrophages, LAMP1⁺ LRO polarization is preserved, but FasL fails to co-accumulate at the cortex, consistent with a terminal trafficking/docking defect. This indicates that ZNRF1 maintains immune balance by licensing a late vesicular step required for FasL to reach the plasma membrane, thereby constraining T-cell responses.

The biological relevance of this checkpoint is underscored by the phenotype of myeloid-specific *Znrf1*^myeΔ^ mice. These animals exhibit age-dependent spontaneous splenomegaly and, upon allosensitization, an elevated CD4/CD8 ratio, expansion of germinal centers, heightened Tfh/IL-21 activity, and increased class-switched alloantibodies—hallmarks of exaggerated humoral immunity. These features mirror aspects of FasL pathway insufficiency described in gld mice (a C-terminal FasL point mutation) and in Fas/FasL signaling defects in humans, which present with lymphoproliferation, splenomegaly, and dysregulated antibody production [22, 23, 34–36]. Macrophages can delete susceptible lymphocytes by expressing surface FasL and crosslinking Fas on target cells [37, 38]; thus, a failure of macrophages to mobilize FasL provides a parsimonious explanation for the in vivo T- and B-cell hyperactivation we observe.

At the mechanistic level, our data pinpoint a post-translational trafficking defect. In RAW264.7 macrophages and in primary BMDMs, total FasL protein increased after LPS, yet surface FasL failed to rise in *Znrf1*-deficient cells in response to LPS or live E. coli. This indicates a selective defect in FasL surface delivery rather than generalized disruption of activation-induced surface receptor/checkpoint expression. Imaging then localized the defect. Upon LPS, control cells formed a cortical accumulation of LAMP1⁺ puncta that co-accumulated with FasL, whereas *sgZnrf1* cells also formed a peripheral LAMP1⁺ annulus, but FasL failed to co-enrich at the cortex, remaining perinuclear with sparse or offset peaks in signal profiles. Thus, ZNRF1 is dispensable for LAMP1⁺ LRO polarization, but required to couple FasL cargo to these peripheral sites for terminal delivery [25, 26].

Vesicle fusion at the plasma membrane is controlled by SNAREs and Sec1/Munc18 (SM) proteins. In hematopoietic secretory lysosomes, Munc18-2 and Syntaxin-3 (Stx3) are key regulators of granule docking/ fusion [28, 31, 32]. Consistent with this paradigm, ZNRF1 loss reduced Stx3 recovery in Munc18-2 co-IPs at baseline and after LPS, without overtly changing inputs (Fig. 3a), and confocal microscopy revealed discrete tri-colocalized cortical puncta of FasL/Munc18-2/Stx3 in control cells that were absent or displaced in *sgZnrf1* (Fig. 3b). Functionally, *Stxbp2* (Munc18-2) knockdown decreased surface FasL, whereas *Syntaxin3* knockdown had a smaller effect in our conditions, placing Munc18-2 as a rate-limiting determinant of FasL delivery. Reconstitution experiments sharpened the mechanism: ZNRF1-WT restored surface FasL and increased Stx3–Munc18-2 association, while ZNRF1(C184A)—despite enhancing complex recovery—failed to rescue surface FasL. We infer a two-tier model whereby ZNRF1 (i) provides a scaffold/stability function that favors Munc18-2–Stx3 assembly and (ii) via its E3 ligase activity, enables productive coupling/priming of FasL-bearing LROs for cortical docking and fusion.

This trafficking defect has measurable functional consequences. In live-cell cocultures, *Znrf1*-deficient macrophages were less cytotoxic to CD3/CD28-activated CD4^+^ T cells over 16 h; T cells displayed sustained survival relative to *sgCtrl*. Using L1210-Fas targets—highly sensitive to Fas-mediated killing—*sgCtrl* effectors induced robust apoptosis across E:T ratios, whereas *sgZnrf1* effectors yielded higher target viability with reduced Annexin V/7-AAD signatures (Fig. 4c, d). Importantly, high-dose LPS priming (500 ng/mL) further potentiated killing by *sgCtrl* but not by *sgZnrf1* effectors, indicating that stronger activation does not bypass the ZNRF1-dependent step. Together, the data support a model in which macrophage FasL, mobilized through a ZNRF1-Munc18-2–Stx3 axis, contributes meaningfully to contraction of T-cell responses alongside the canonical T- and NK-cell FasL pathways [24, 37, 38].

These findings align with, and extend, the concept of secretory lysosomes (lysosome-related organelles, LROs) that store and deploy immune effectors [39, 40]. In T and NK cells, FasL resides with granzymes/perforin in LROs and is mobilized to the surface upon activation [3, 25, 41]. Our macrophage data suggest a parallel LRO pathway in which FasL shares trafficking components with mast-cell degranulation (Munc18-2/Stx3) [30] and possibly with other myeloid secretory routes. The lack of an effect on the Munc18-2–Syntaxin-11 interaction (data not shown) [42, 43] argues that distinct syntaxin partnerships specify cargo-selective routes, with the FasL pathway relying on Stx3 rather than Stx11. Analogies from platelets (Munc18-2-dependent granule release) and human immunopathology (Munc18-2 mutations in familial hemophagocytic lymphohistiocytosis type 5) support a broader principle: SM/SNARE modules act as bottlenecks for secretory LRO exocytosis across hematopoietic lineages [44, 45]. In this context, our identification of ZNRF1 as an upstream regulator of the Munc18-2–Stx3 module raises the possibility that reduced ZNRF1 activity could compromise the efficiency of Munc18-2-dependent LRO exocytosis and thereby contribute to FHL-like immune dysregulation. Nevertheless, FHL5 is genetically defined by loss-of-function STXBP2 mutations and typically involves broad defects in cytotoxic lymphocyte degranulation; thus, our current data do not establish a direct role of ZNRF1 in human FHL5 pathogenesis. Instead, we propose that ZNRF1 may represent a potential modifier or parallel regulatory node that tunes Munc18-2–Stx3 function in specific cellular contexts (including myeloid cells), a hypothesis that could be tested using patient-derived cells and primary NK/CD8 T-cell degranulation assays alongside trafficking readouts.

We note several limitations that inform future work. First, neutralization with anti-FasL antibodies yielded inconclusive results, likely due to Fc receptor-mediated internalization by macrophages and potential binding to both effectors and targets, confounding functional blockade [46]. Fc-silent antibodies, Fas-Fc decoys, or genetic FasL ablation in effectors would provide cleaner tests of FasL dependence. Second, while our imaging identifies cortical miscoupling of FasL from LAMP1⁺ compartments in *sgZnrf1*, it does not yet resolve the molecular substrates of ZNRF1. Identifying ZNRF1 ubiquitination targets within the Munc18-2/Stx3/SM–SNARE/Tether network—or on Rab/effector components—will be crucial. Third, preliminary observations suggest a broader impact of ZNRF1 on secretory-lysosome–positive routes, including MHC class II trafficking under IFN-γ (data not shown), consistent with links between MHC class II vesicles and myeloid LROs [47]; these pathways merit systematic dissection.

Collectively, our findings identify ZNRF1 as a macrophage-intrinsic checkpoint that enforces immune contraction by ensuring surface delivery of FasL and facilitating Fas-dependent apoptosis of activated lymphocytes. Given the clinical links between Fas/FasL dysfunction and lymphoproliferation, the ZNRF1–Munc18-2–Stx3 pathway emerges as a mechanistic node with translational potential; however, because ZNRF1 also regulates broader innate programs, any intervention will require cell-type- and context-specific targeting.

## Conclusions

ZNRF1 is essential for terminal trafficking and surface exposure of FasL in macrophages. Loss of ZNRF1 leaves LAMP1⁺ LRO polarization intact but decouples FasL cargo from cortical docking sites, correlating with diminished Munc18-2–Syntaxin-3 complex functionality. ZNRF1 contributes both a scaffold role that stabilizes the fusion complex and an E3 ligase–dependent activity that enables productive coupling/priming of FasL vesicles. Functionally, ZNRF1 deficiency impairs FasL-mediated apoptosis of activated T cells and FAS-expressing targets, aligning with splenomegaly, altered CD4/CD8 ratios, expanded GC/Tfh-IL-21 programs, and enhanced alloantibody production in vivo. These findings identify a macrophage FasL trafficking checkpoint that is critical for immune contraction and suggest that modulating ZNRF1 or its downstream fusion machinery could provide therapeutic leverage in disorders of immune hyperactivation.

## Supplementary information

Supplementary Data

Original Data

checklist

Supplementary File

## Data Availability

The data supporting the findings of this study are available from the corresponding author upon reasonable request.
