# Supplementary material for: ZNRF1 deficiency disrupts Fas ligand trafficking and immune balance
Source: Cell Death Dis. 2026 Mar 28;17(1):422. doi: 10.1038/s41419-026-08566-8 (PMC13149863; doi:10.1038/s41419-026-08566-8)
Supplement: Supplementary file 1 — Supplementary Data [file 41419_2026_8566_MOESM1_ESM.docx]

**Supplementary Figures
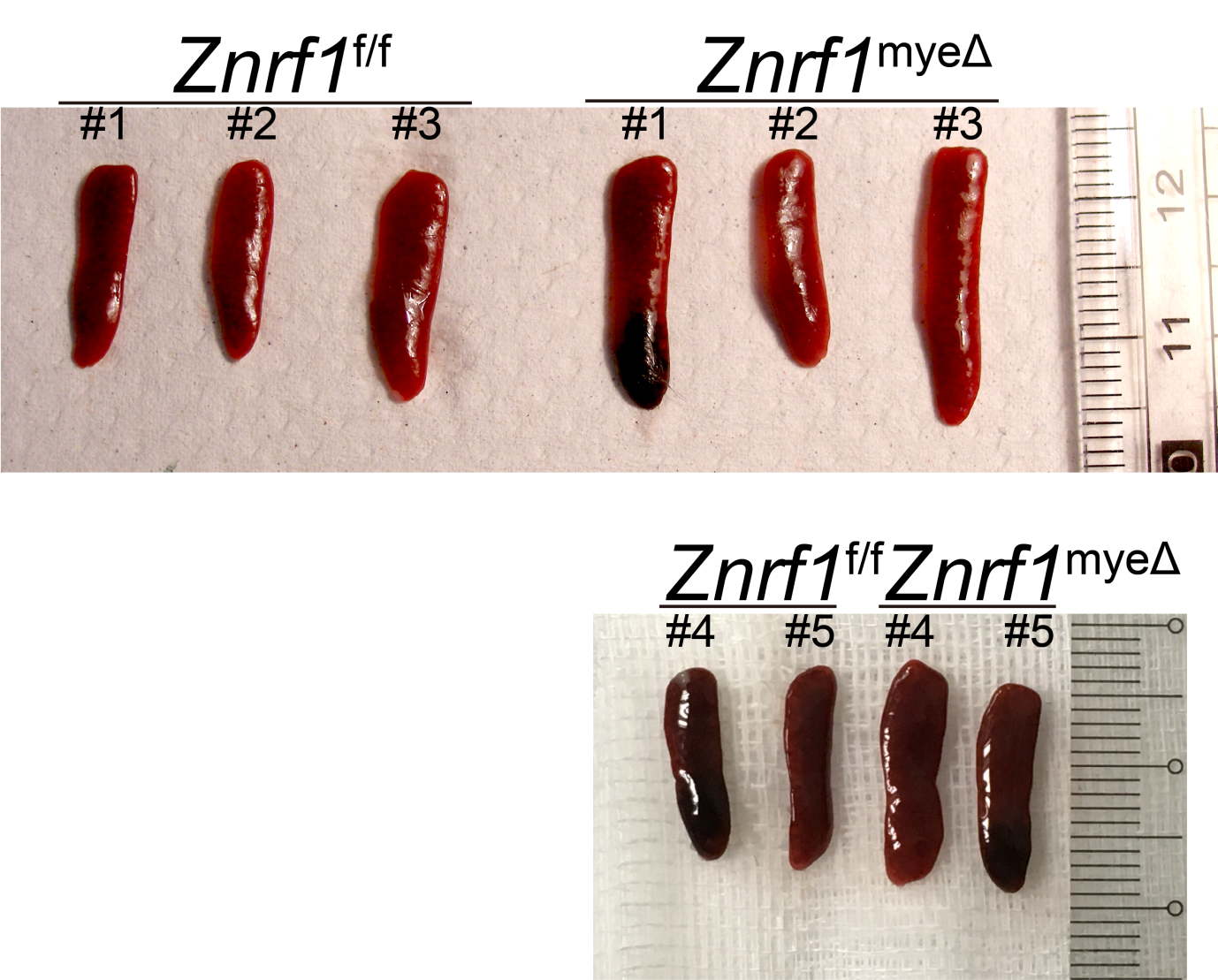
**

**Fig. S1. Gross spleen appearance in the baseline cohort.**Representative photographs of spleens from sham-treated 6–8-week-old *Znrf1*^f/f^ and *Znrf1*^myeΔ^ mice (all animals shown; n = 5 per genotype). Quantification of spleen index for the same cohort is provided in Fig. 1b.


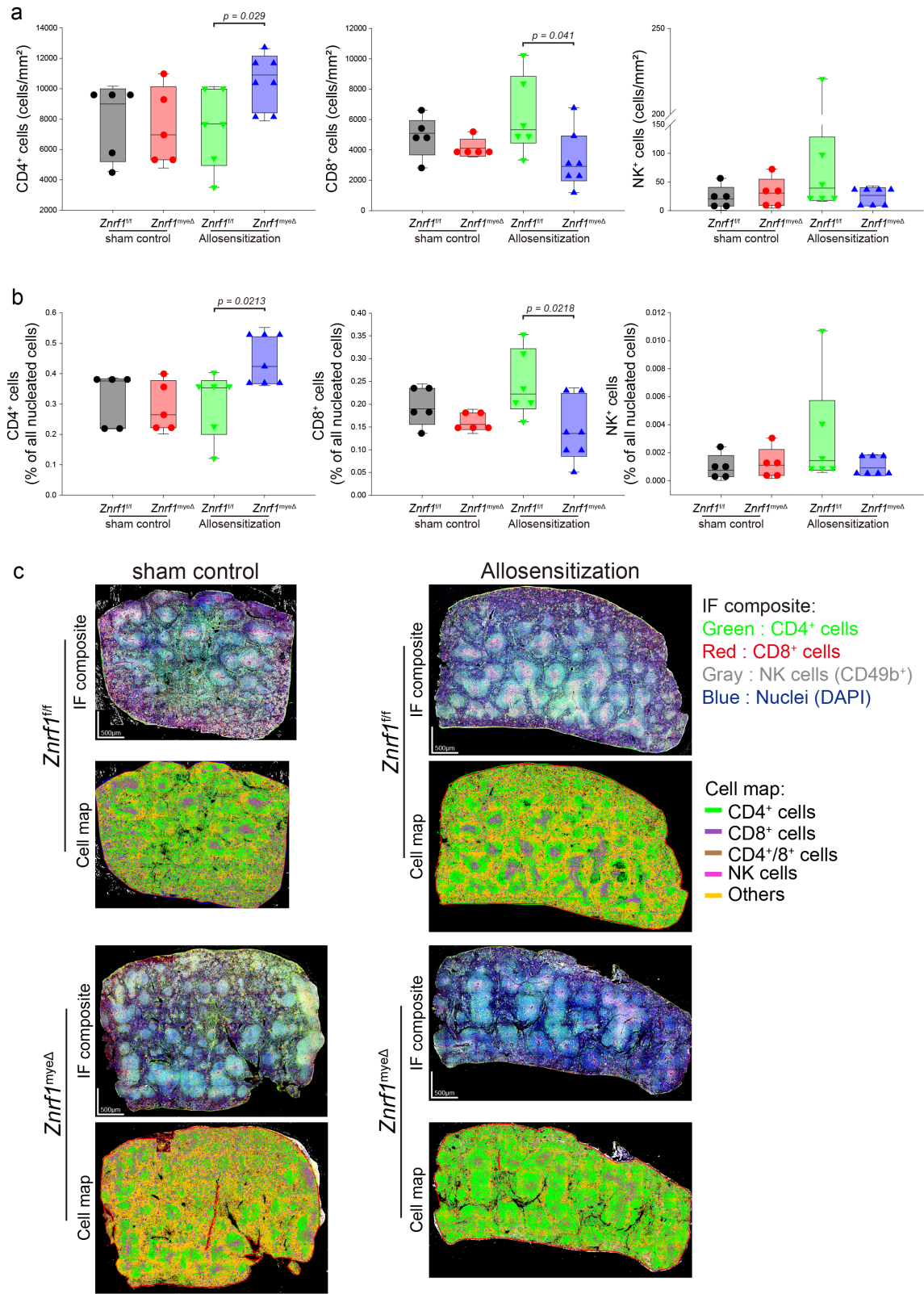


**Fig. S2. Panoramic whole-spleen immunofluorescence quantification of CD4⁺ and CD8⁺ T cells and NK cells in *Znrf1*^f/f^ and myeloid-specific *Znrf1*-deficient (*Znrf1*^myeΔ^) mice under sham control and after allosensitization.**
(a) Cell densities (cells/mm²) of CD4⁺ T cells, CD8⁺ T cells, and NK cells (CD49b⁺) quantified across the entire spleen section from panoramic immunofluorescence images.
(b) Frequencies of CD4⁺ T cells, CD8⁺ T cells, and NK cells expressed as a percentage of all DAPI⁺ nucleated cells in the same sections. (c) Representative whole-spleen panoramic immunofluorescence composites and corresponding cell maps. Immunofluorescence composite images show CD4 (green), CD8 (red), NK cells/CD49b (gray), and nuclei (DAPI; blue). Cell maps were generated by image-based segmentation and marker-based classification, displaying CD4⁺ cells (green), CD8⁺ cells (purple), CD4⁺/CD8⁺ double-positive cells (brown), NK cells (pink), and other cells (yellow). Scale bars, 500 µm. Each symbol represents one mouse. Box-and-whisker plots show the median (center line), interquartile range (box), and minimum to maximum values (whiskers). Statistical significance was assessed by one-way ANOVA; exact *p* values are shown on the plots.

**
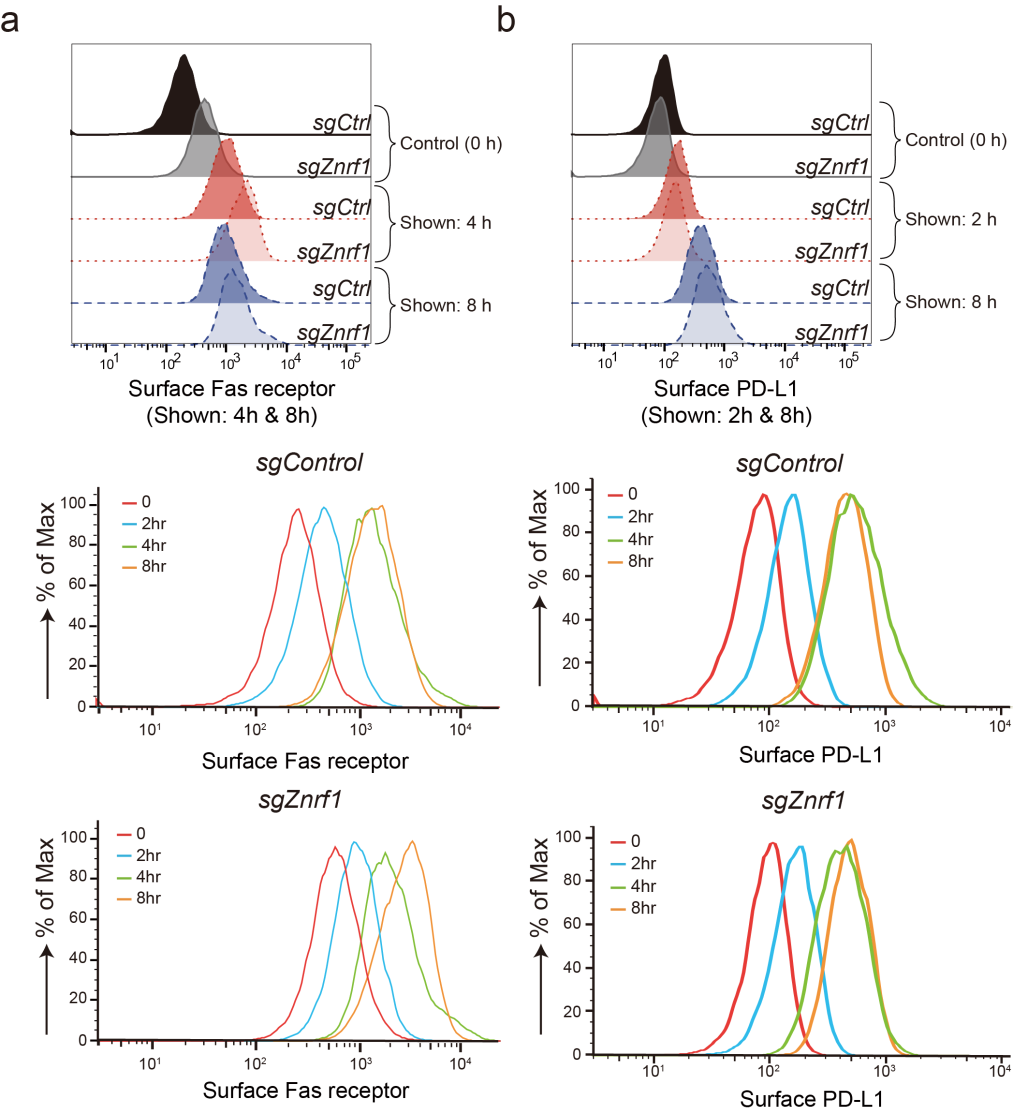
**

**Fig. S3.** **Surface expression of Fas receptor (CD95) and PD-L1 in macrophages after LPS stimulation.**

(a) *sgCtrl* and *sgZnrf1* RAW264.7 macrophages were stimulated with LPS (100 ng/mL) for the indicated times (0, 2, 4, and 8 h), and surface Fas (CD95) was analyzed by flow cytometry. For clarity, the representative stacked histograms (top) show the unstimulated control together with 4 h and 8 h, whereas the overlaid kinetics plots (bottom) show all time points. (b) *sgCtrl* and *sgZnrf1* RAW264.7 macrophages were stimulated with LPS (100 ng/mL) for the indicated times (0, 2, 4, and 8 h), and surface PD-L1 was analyzed by flow cytometry. For clarity, the representative stacked histograms (top) show the unstimulated control together with 2 h and 8 h, whereas the overlaid kinetics plots (bottom) show all time points. Data are representative of three independent experiments.


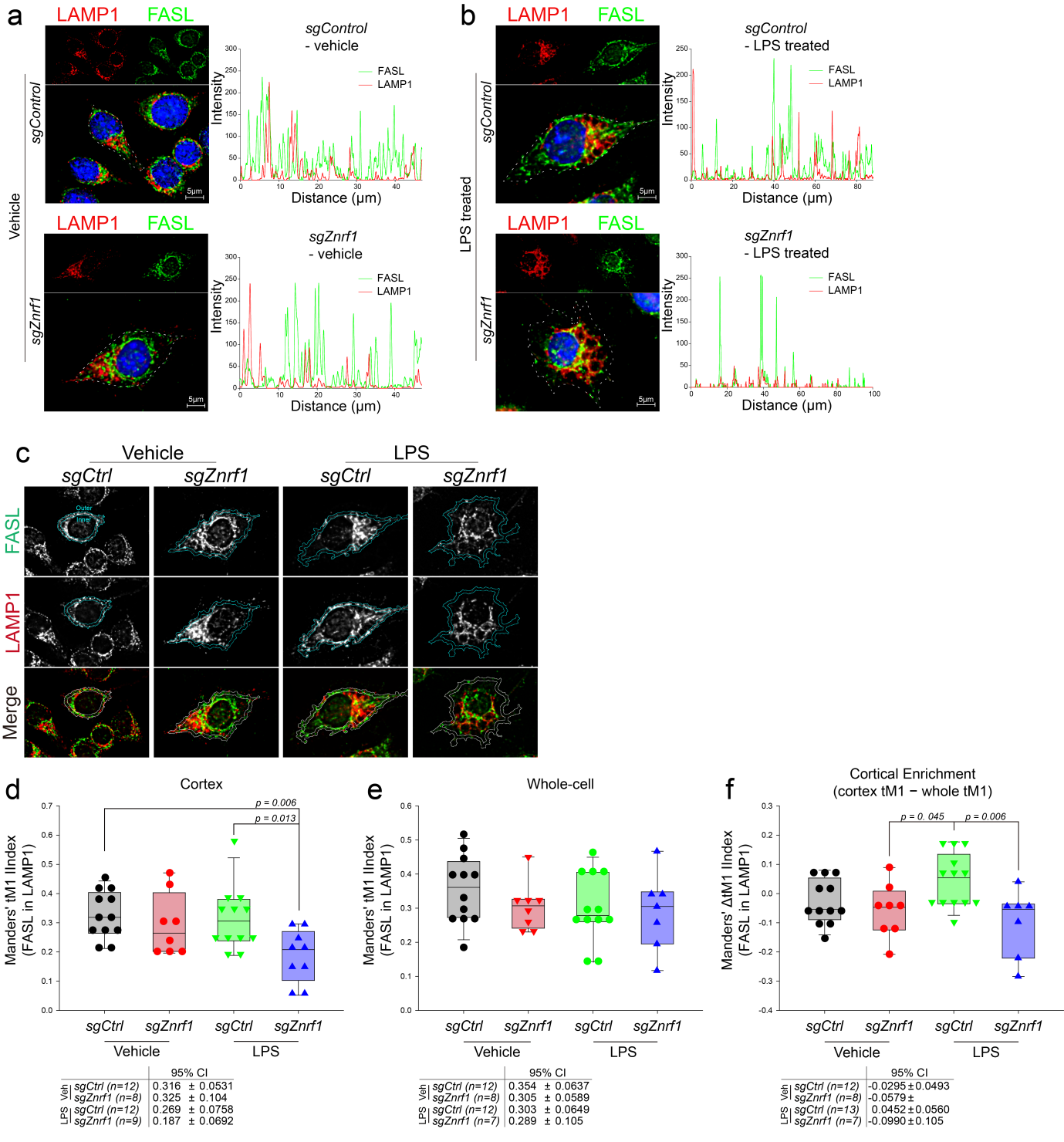


**Fig. S4. Differential LAMP1–FasL colocalization in ZNRF1-deficient macrophages at resting and after LPS stimulation**
(a) RAW264.7 macrophages (*sgControl* and *sgZnrf1*) were fixed and immunostained for LAMP1 (red) and FASL (green); nuclei counterstained with DAPI (blue). Images represent maximum-intensity projections of confocal z-stacks (all optical sections merged). Dashed white lines mark the cell boundaries. Right, representative signal intensity profiles of FASL (green) and LAMP1 (red) across the cell. Scale bars, 5 µm. (b) Cells were stimulated with LPS (100 ng/mL, 20 h). Images are also maximum-intensity projections of confocal z-stacks. Signal intensity profiles are shown on the right. (c) Cyan outlines indicate the outer whole-cell contour and a 1-µm inner inset. The cortical ring ROI is the 0–1 µm annulus between the outer and inner contours; the whole-cell ROI corresponds to the outer contour. Single-channel grayscale and merged images are shown for vehicle-treated and LPS-treated cells of each genotype. (d) Costes-thresholded Manders’ tM1 measured within the cortical ring ROI for vehicle-treated and LPS-treated *sgControl* and *sgZnrf1* cells. Boxplots display median and IQR with Tukey 1.5×IQR whiskers; dots are single cells. n (cells) and mean ± 95% CI for each group are listed below the panel. n denotes the number of cells passing ROI-specific QC for the cortical analysis. (e) Same analysis as in (d) but performed within the whole-cell ROI. Box/whisker/points as in (d); n and mean ± 95% CI listed below. n denotes the number of cells passing QC for the whole-cell analysis; n may differ from (d) due to ROI-specific QC. (f) For each cell, ΔtM1 = tM1(cortex) − tM1(whole-cell). Box/whisker/points as in (d); n and mean ± 95% CI listed below. Only cells passing QC in both ROIs were included (paired per cell).

**
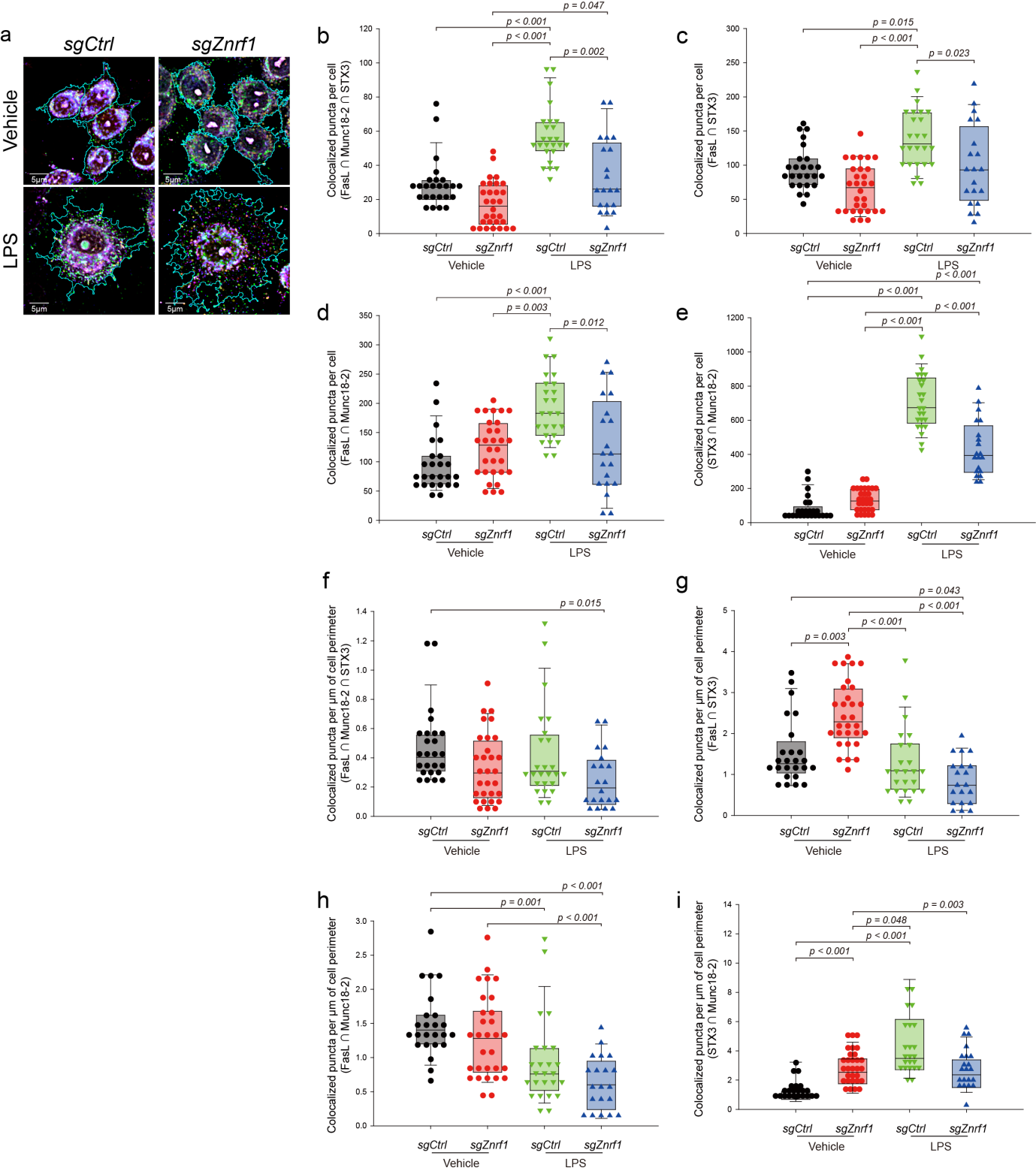
**

**Fig. S5. Object-based colocalization of FasL, Stx3 and Munc18-2 within whole-cell ROIs.**

(a) Representative maximum-intensity projections (FasL, Stx3, Munc18-2) with whole-cell ROIs (cyan; filopodia included). Orange circles mark tri-colocalized puncta. Scale bars, 5 μm.

(b–e) Per-cell counts of colocalized puncta for the indicated channel relations: (b) FasL∩Stx3∩Munc18-2 (triple), (c) FasL∩Stx3, (d) FasL∩Munc18-2, (e) Munc18-2∩Stx3. Here “A∩B” denotes A-channel puncta having ≥1 B-channel neighbor within Dmax; “A–B (paired)” denotes one-to-one A/B assignments within Dmax. (f–i) Perimeter-normalized densities (puncta per μm of the same cell perimeter ROI) for the same relations as in b–e: (f) FasL∩Stx3∩Munc18-2 per μm, (g) FasL∩Stx3 per μm, (h) FasL∩Munc18-2 per μm, (i) Munc18-2∩Stx3 per μm. Box-and-whisker plots: center line = median; box = IQR (Q1–Q3); whiskers = 10–90th percentiles; all points shown. Exact P values were annotated. ColocQuant parameters: σ (Ch1/Ch2/Ch3) = 1.4/1.4/1.8; thresholds = 2.1/2.3/2.3; Dmax = 2 px


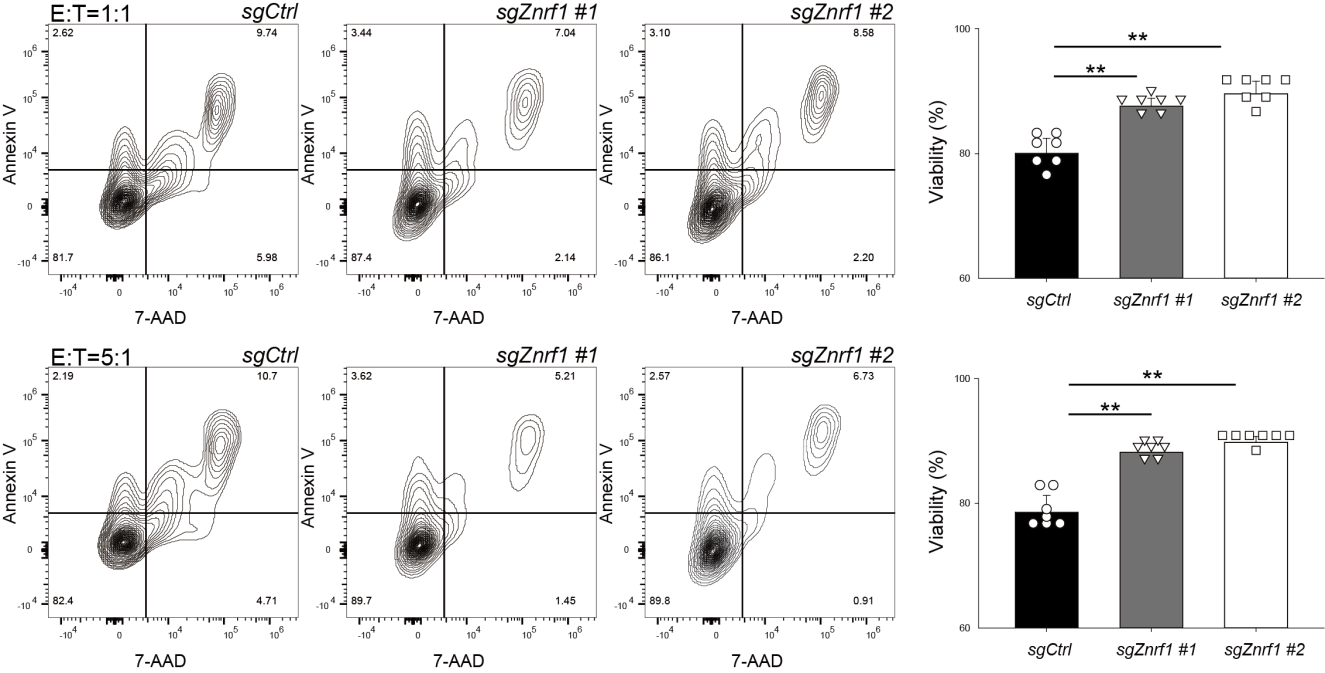


**Fig. S6.** **High-dose LPS enhances cytotoxicity of control macrophages but not ZNRF1-deficient macrophages.** CFSE-labeled L1210-Fas cells were co-cultured with *sgControl* or *sgZnrf1* RAW264.7 macrophages at effector:target (E:T) ratios of 1:1 and 5:1. Macrophages were primed with high-dose LPS (500 ng/mL), and target-cell viability was assessed after co-culture by annexin V/7-AAD staining and flow cytometry. Representative contour plots and summary bar graphs are shown. Data are from two independent experiments (total n = 7 per group). **p < 0.01.
