# Supplementary material for: ZNRF1 deficiency disrupts Fas ligand trafficking and immune balance
Source: Cell Death Dis. 2026 Mar 28;17(1):422. doi: 10.1038/s41419-026-08566-8 (PMC13149863; doi:10.1038/s41419-026-08566-8)

**Uncropped Western Blots**

Figure 2a


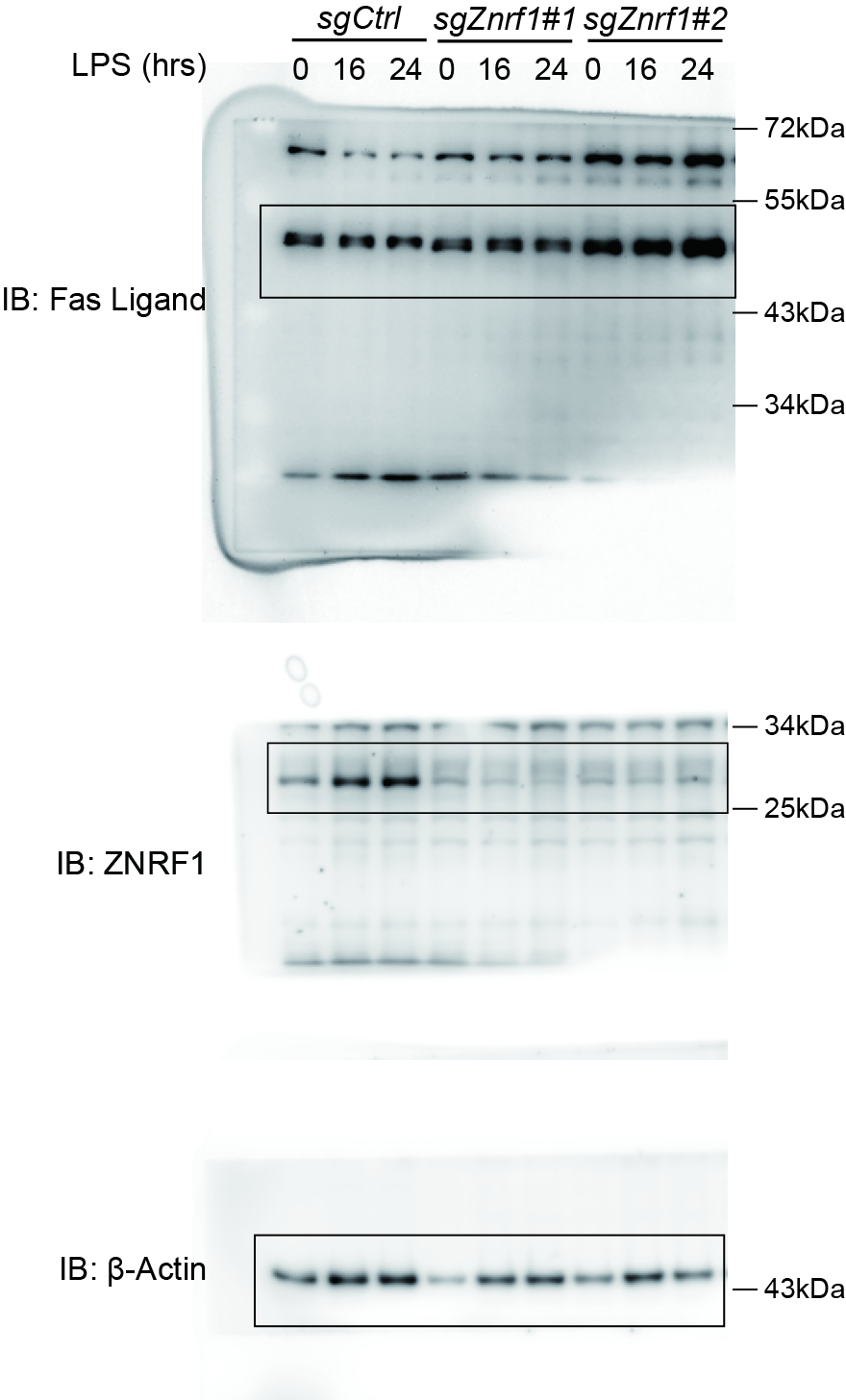


**Uncropped Western Blots**

Figure 2d


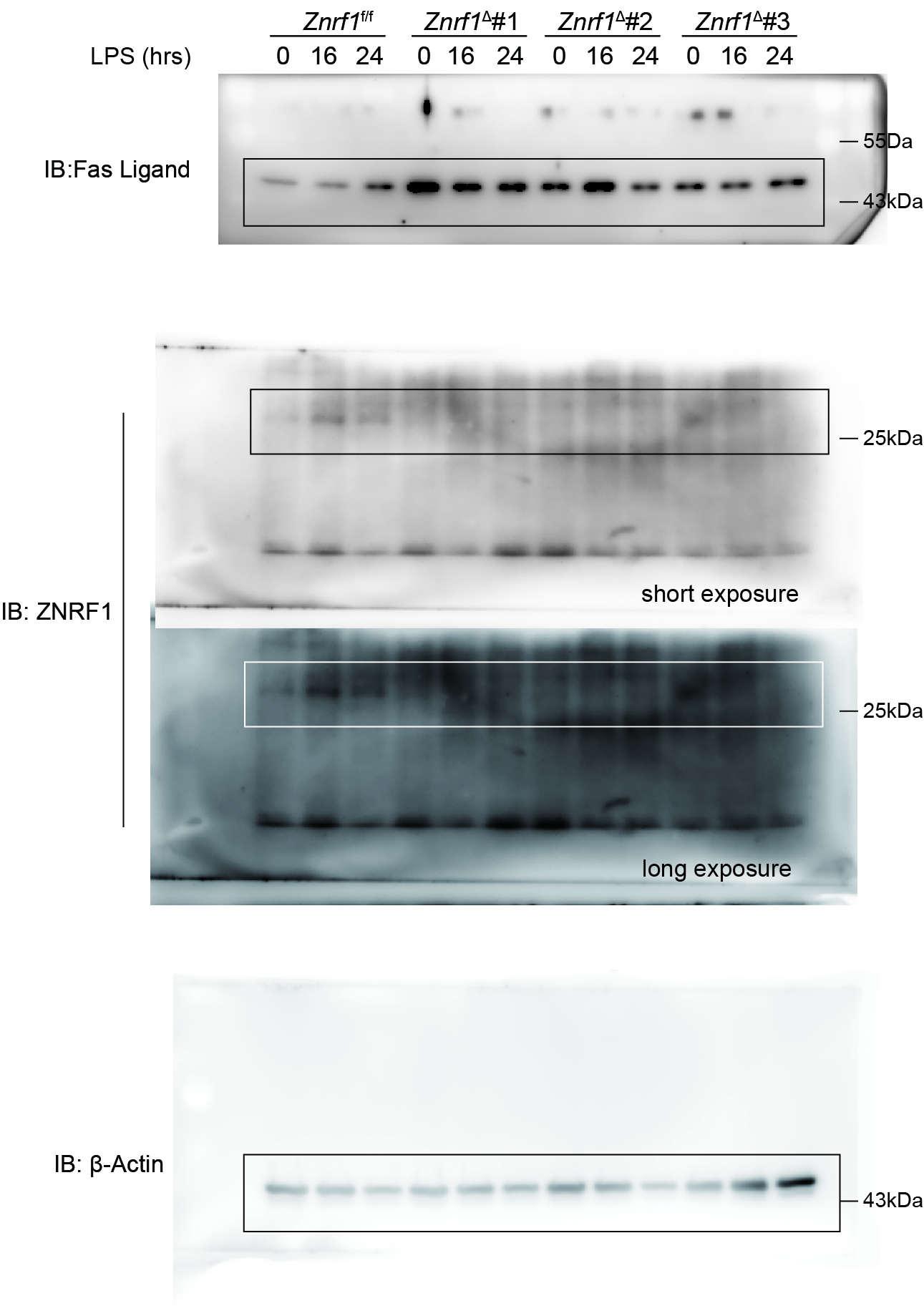


**Uncropped Western Blots**

Figure 3a


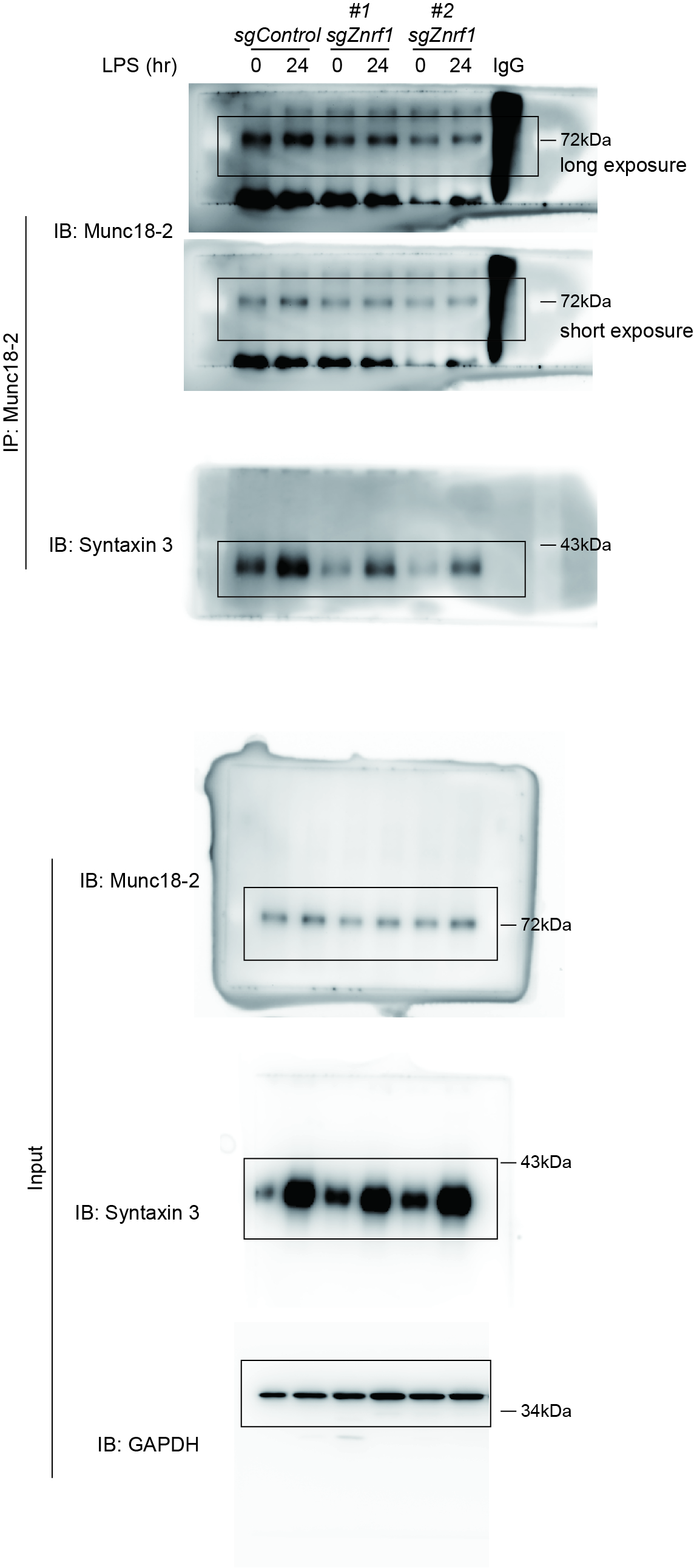


**Uncropped Western Blots**

Figure 3d


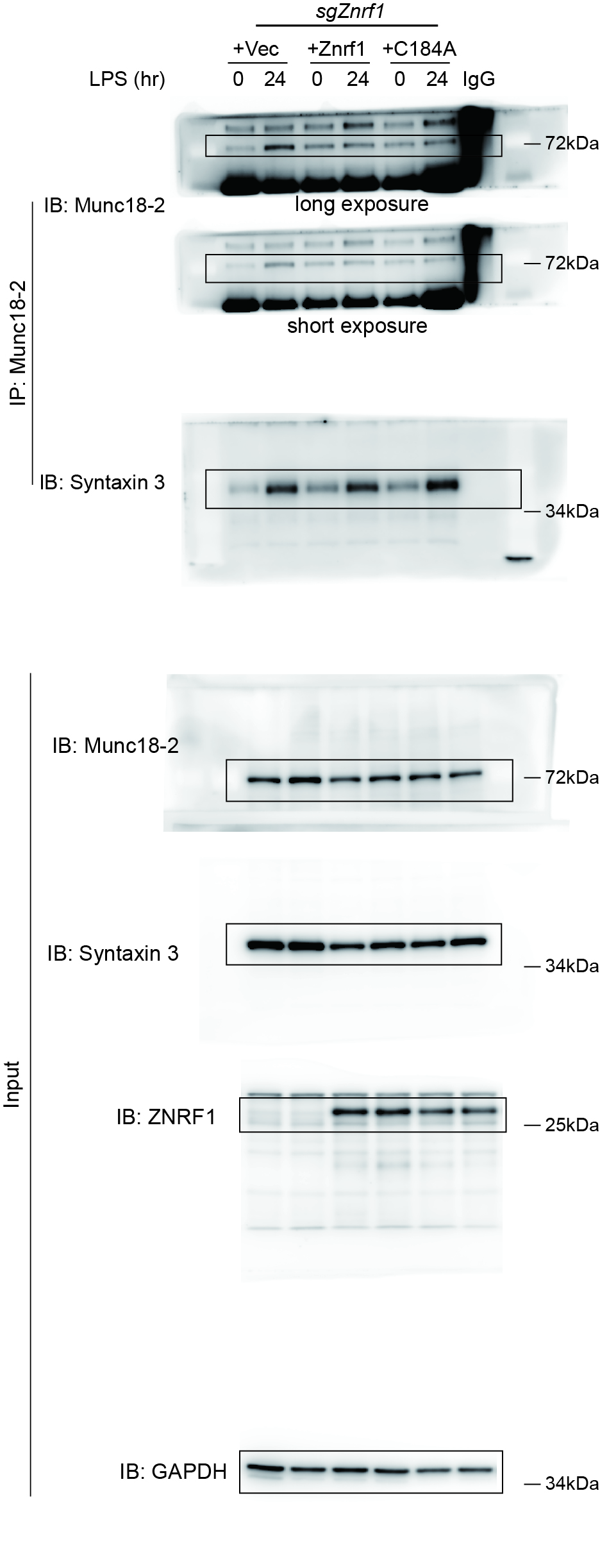

Supplement: Supplementary file 2 — Original Data [file 41419_2026_8566_MOESM2_ESM.docx]
