# Supplementary material for: ZNRF1 deficiency disrupts Fas ligand trafficking and immune balance
Source: Cell Death Dis. 2026 Mar 28;17(1):422. doi: 10.1038/s41419-026-08566-8 (PMC13149863; doi:10.1038/s41419-026-08566-8)
Supplement: Supplementary file 4 — Supplementary File [file 41419_2026_8566_MOESM4_ESM.docx]

**Supplementary File**

**Image analysis workflows for colocalization quantification**

*Image preprocessing*

Confocal z-stacks were acquired using identical imaging settings across experimental conditions within each comparison. For analysis, images were subjected to uniform background subtraction, converted to 16-bit format, and rendered as maximum-intensity projections. All quantification was performed on projected images using the same preprocessing workflow across groups. Cell-by-cell analysis was based on manually defined whole-cell contours, with cell-cell contact regions, debris, and truncated border cells excluded

*Intensity-based colocalization of FasL and LAMP1*

Intensity-based colocalization of FasL and LAMP1 was performed in FIJI/ImageJ using the Coloc2 plugin. FasL was assigned to Channel 1 (FasL-488) and LAMP1 to Channel 2 (LAMP1-594), and colocalization was quantified using Costes automatic thresholding.

For each cell, an outer whole-cell contour (Outer) was manually drawn to encompass the full cell body, including major peripheral protrusions while excluding neighboring cells and debris. An inner contour (Inner) was then generated by an inward offset of 1 µm from the outer contour. The cortical ROI was defined as the annular belt between these contours, corresponding to the XOR of Outer and Inner and representing a 0–1 µm peripheral ring. The whole-cell ROI corresponded to the Outer contour.

The primary readout was Manders’ tM1, defined as the fraction of FasL signal overlapping LAMP1 signal after Costes thresholding. Manders’ tM1 was calculated separately in the cortical ROI and the whole-cell ROI. Cortical enrichment was defined on a per-cell basis as ΔtM1 = tM1(cortex) − tM1(whole-cell), where positive values indicate relative enrichment of FasL-LAMP1 colocalization at the cell cortex.

Only ROIs satisfying all predefined quality-control criteria were retained: (i) Costes P ≥ 0.90; (ii) thresholds that did not collapse to zero or to the maximal gray value; (iii) absence of degenerate threshold behavior; and (iv) absence of obvious segmentation errors or contamination by adjacent cells or debris. ΔtM1 was calculated only when both the cortical ROI and the whole-cell ROI passed quality control for the same cell.

*Object-based triple colocalization of FasL, Syntaxin-3, and Munc18-2*

Object-based triple colocalization of FasL (Ch1), Syntaxin-3/Stx3 (Ch2), and Munc18-2 (Ch3) was performed in FIJI/ImageJ using a ColocQuant/ColocJ-based workflow on maximum-intensity projected images.

Puncta were detected after Laplacian-of-Gaussian (LoG) spot enhancement using channel-specific thresholds defined as:

Threshold = mean image intensity + c·SD

The detection parameters were as follows: LoG σ = 1.4 px for Ch1 (FasL), 1.4 px for Ch2 (Stx3), and 1.8 px for Ch3 (Munc18-2); threshold coefficients c = 2.1 for Ch1, 2.3 for Ch2, and 2.3 for Ch3.

Pairwise and triple-colocalized puncta were defined by mutual nearest-neighbor assignment with a maximum linking distance of Dmax = 2 pixels. Two puncta in different channels were considered pair-colocalized only if each punctum was the other’s nearest neighbor within Dmax. Triple-colocalized puncta were defined when puncta from all three channels satisfied this assignment criterion through the corresponding pairwise relationships.

Readouts included total counts of pairwise-colocalized puncta, total counts of triple-colocalized puncta, and perimeter-normalized densities of colocalized puncta (puncta/µm). All densities were normalized using the same manually defined whole-cell contour used for ROI-based analysis.

*Statistical analysis*

Normality and equal variance were assessed using the Shapiro-Wilk and Brown-Forsythe tests, respectively. Group comparisons were performed using one-way ANOVA or Kruskal-Wallis testing, as appropriate, followed by Holm-Sidak, Tukey, or Dunn’s post hoc tests for prespecified contrasts. For ΔtM1, enrichment relative to zero was additionally evaluated using the Wilcoxon signed-rank test.
